# Refugee Youth and Transition to Further Education, Training, and Employment in Australia: Protocol for a Mixed Methods Study

**DOI:** 10.2196/12632

**Published:** 2019-07-31

**Authors:** Tahereh Ziaian, Emily Miller, Helena de Anstiss, Teresa Puvimanasinghe, Maureen Dollard, Adrian Esterman, Helen Barrie, Tamara Stewart-Jones

**Affiliations:** 1 School of Psychology, Social Work & Social Policy Magill Campus University of South Australia Magill Australia; 2 School of Nursing & Midwifery City East Campus University of South Australia Adelaide Australia; 3 Hugo Centre for Migration and Population Research University of Adelaide Adelaide Australia; 4 Multicultural Youth South Australia Adelaide Australia

**Keywords:** refugee, youth, education, employment, mixed-methods, Australia

## Abstract

**Background:**

Young people with refugee experiences are widely acknowledged as encountering multiple disadvantages that affect their school completion and retention, university entry, and subsequent employment. This paper discusses the rationale for and protocol of a mixed methods investigation focusing on improving education and employment outcomes among refugee background youth aged 15 to 24 years from three focus regions: the Middle East (Afghanistan, Iran, Iraq, Syria), South Asia (Nepal, Bhutan, Myanmar/Burma, Pakistan) and Africa (Sudan, South Sudan, Liberia, Ethiopia, Somalia, DR Congo).

**Objective:**

The rationale of the project is to identify the facilitators and barriers to successful transition from school to further education and employment; investigate participant awareness of support systems available when faced with education and employment difficulties; redress the disadvantages encountered by refugee background youth; and bridge the gap between research, policy, and practice in relation to social inclusion and participation.

**Methods:**

The study involves collecting survey data from 600 youth followed by individual interviews with a subset of 60 youth, their parents/primary caregivers, and their teachers. A cross-sectional survey will assess facilitators and barriers to successful transition from school to further education and employment. Individual interviews will provide context-rich data on key issues relevant to education and employment outcomes.

**Results:**

The study began in 2016 and is due for completion by the end of 2019. The quantitative survey has been conducted with 635 participants and was closed in March 2019. The qualitative interview stage is ongoing, and the current total in April 2019 is 93 participants including educators, youth, and family members of the youth. Analysis and presentation of results will be available in 2020. Some preliminary findings will be available during the late half of 2019.

**Conclusions:**

This project will contribute new and unique insights to knowledge in relation to key factors influencing education and employment outcomes among refugee youth. This research will enable effective planning for the needs of some of Australia’s most disadvantaged and marginalized young people, leading to a sustainable improvement in the education and employability of young refugees.

**International Registered Report Identifier (IRRID):**

DERR1-10.2196/12632

## Introduction

### Background

A large proportion of humanitarian arrivals in Australia are children who receive their education in their new home country [1]. They may face unique challenges that place them at increased risk of poor education and employment outcomes [2-5] such as limited English language skills [6,7]; difficulty navigating the education, training, and employment systems; limited or interrupted former education; psychosocial problems associated with premigration and postmigration stressors; lack of support at school and home; and racism and discrimination [6,8-10]. Refugee intakes can diversify and increase the skill level of Australia’s population [2,11] with long-term positive economic impacts for Australia [1,12]. However, the effect of multiple disadvantages on school completion and retention rates, university entry, and subsequent employment for refugee youth is widely acknowledged [2,3,5,7,13].

Australia is a multicultural nation, with 49% of all Australians either born overseas or having at least one parent born overseas [14]. Of recent migrants, 76% were born in other than English-speaking countries and 91% were aged 15 to 44 years on arrival [14]. Refugees are a growing group in the broader migrant population, with more than 800,000 arriving since 1945, or 13,750 arrivals each year [1,11,15]. Young people account for 25% of all youth aged 12 to 24 years in Australia and about 50% of Australia’s humanitarian intake [11,14,16].

There is consensus that recently arrived refugee youth face a different and unique set of challenges that place them at increased risk of education and employment challenges outside the experience of youth in the general population [7,17]. Similar to migrant youth, young refugees must locate and connect themselves within a new cultural environment [18] and work through life cycle challenges (eg, identity development during adolescence) but unlike migrant youth, they must also try to find a sense of safety and security within fractured families and communities while addressing consequences of experiencing and/or witnessing past traumatic events during armed conflict, organized violence, and prolonged displacement [4]. Additionally, many refugee youths are not accessing support services that could increase their prospects for successful integration [19,20] and improved employment opportunities.

Schools have the potential to not only provide educational opportunities but, more importantly, offer a means of integration into the host society [3]. School is a prime location for refugee youth to begin building a new sense of civic identity [4,21] and belonging [7,20]. Education is crucial in establishing routines and giving a sense of normality and hope for children’s futures. School can be a place of security when all other things may seem to be in confusion [22].

Although school has the potential for positive outcomes and experiences, structural, cultural, and language barriers place refugee students on an unequal footing with their Australian-born peers. The interplay between structure, culture, and agency is a framework within which the nature of power structures and experiences of school may be interrogated [23]: the significance of a student’s ethnicity, for example, is determined by a range of factors including institutional bias, school policies, or the agency of individuals to be flexible within a school’s hierarchical systems [24].

Adding further pressure are parental expectations that these youth enter highly competitive professions such as medicine, engineering, and dentistry [6]. Most refugee parents suffer a loss of role and status with migration [25,26]. Many are living on welfare payments or working in unskilled, low-paid, and low-status jobs [27], which for the more educated and affluent represent a marked change from their former circumstances. For their part, many young people feel their parents do not understand how difficult it is to excel in a new and unfamiliar education and employment environment [6].

There is a recurring issue of language as a barrier to accessing health, employment, and education services [28,29]. English language challenges and limited understanding of school structure and processes may prevent many parents from helping students with studies or attending parent interviews and other school activities designed to support children in their studies [30].

People with a refugee background may be a part of a culturally identifiable group; however, it is vital to acknowledge that these groups are, in turn, made up of unique individuals with diverse contextual needs [21]. Supporting diversity with the aim of successful settlement and integration of refugees is a critical issue for host societies [4], and engaging with the labor market is pivotal to this [1,13]. The settlement of refugees is more difficult in virtually all aspects than the settlement of other migrants, and employment outcomes are considerably poorer than for other migrants [2].

Education has the potential to broaden opportunities for employment and increasing individuals’ capacity to make choices about their career path. Although research has been undertaken on the educational experiences of young refugees in Australia and elsewhere [4,7,20,25,31-33], studies of actual education and employment outcomes are scarce, yet these are crucial stages through which individuals have opportunities for development into fully participating, productive Australian citizens.

### Aim

The main aim of this project is to investigate education and employment outcomes among refugee youth aged 15 to 24 years from three focus regions—the Middle East (Afghanistan, Iran, Iraq, Syria), South Asia (Nepal, Bhutan, Myanmar/Burma, Pakistan) and Africa (Sudan, South Sudan, Ethiopia, Liberia, Somalia, DR Congo)—with a view to influencing education, training, and employment policy and practice.

The following objectives will be employed for meeting project aims:

- Identify facilitators and barriers to successful transition from school into further education and employment
- Map out support systems accessed by youth who are experiencing education- and employment-related difficulties
- Investigate the extent of youth and family awareness of available education, training, and employment pathways
- Bridge a gap between research, policy, and practice in relation to the social inclusion and participation of refugee youth in educational settings
- Propose evidence-based recommendations for policy and decision makers in the education, training, and employment sectors
- Respond to social justice concerns by providing new insights into potential improvements to the long-term employment opportunities of refugee youth

### Theoretical Framework

In the absence of refugee youth visibility in policy and research, there is no one theoretical framework appropriate to help us address the significant educational disadvantages confronting refugee youth and their migration experiences, educational participation, adjustment, and integration. This research is innovative and important because it brings together two frameworks to generate an integrated conceptual framework (integrated immigration model) to help understand refugee youth educational participation, adjustment, and integration (cultural, social, and economic). First, the segmented assimilation model combines elements of both the assimilation and ethnic disadvantage perspectives by recognizing the diversity of settlers’ experience [34]. Second, the advocacy/transformative conceptual framework, marked by the conscious inclusion of groups that are generally excluded from mainstream society, allows participants to benefit both during the research process and afterward when findings are used to bring changes in action or policy [35,36]. This theoretical lens sees action as an important outcome of research and provides methodological guidance for researchers working in the interest of social justice in culturally complex communities to improve unsatisfactory social conditions and outcomes for marginalized population groups.

The migration experience is clearly impacted by the experience and knowledge that settlers bring with them, the context into which they arrive, and the responsiveness of the host community [1]. We expect the integration pathways to be affected by these contextual factors. Our integrated approach argues that some settlers experience structural barriers that limit their access to employment and other opportunities while others experience upward mobility. Our approach also emphasizes multiple pathways to incorporation, and the policy emphasis is on identifying the contextual, structural, and cultural factors that separate successful incorporation from unsuccessful integration [1,34].

The main aim of the study is to investigate education and employment outcomes among refugee youth aged 15 to 24 years from three focus regions. Findings from the study will contribute new and unique insights and knowledge relating to key factors influencing further education and employment outcomes among refugee youth in Australia.

## Methods

### Selection Criteria

The survey component will target youth aged 15 to 24 years who have migrated, or whose parents have migrated, to Australia since 2006 and are studying at the time of participation. The criteria for ethnicity will be self-ascribed ethnic origin. Families with more than one child will be given the option of having up to two of their eligible youth participate in the study. The method of selection will be the birthday technique (ie, two youths with birthdays closest to the date of the interview will be selected).

Time of arrival is restricted to the last 10 years because the initial and middle stages of migration and resettlement present particular adjustment problems [37] that are the focus of this study. The interview stage will only accept consenting participants (with parental consent for those under age 18 years) who additionally consent to interviews of their caregivers and teachers.

### Sample Size

We assume approximately 60,000 to 70,000 refugee youth aged 15 to 24 years are resident in Australia [15,16]. With regard to the quantitative component of this study, a sample of 600 would provide the following accuracy with 95% confidence for any question with an expected frequency response of 50%: overall (±4%), for each gender (±5.5%), for each migration region (±7%), and for gender within migration region (±10%).

The qualitative component will purposively target 60 youth (20 youth for each of the target migration regions) along with their parents/primary caregivers (60) and their teachers (60) from the larger sample allowing 10% of the entire survey group to be represented in the qualitative stage of the study.

See Multimedia Appendix 1 for a report on the demographic profile of the South Australian refugee youth population in 2019.

### Sampling Method

We will use a combination of convenience and snowball sampling to recruit the research participants. Convenience and snowball sampling are generally used when the desired characteristics of the sample are uncommon, when the target population is difficult to access, and when nonprobability sampling methods are not possible [38], all of which apply to refugee populations.

### Recruitment

The sample of participating youth will be drawn from youth with refugee backgrounds who are studying at the time of participation. Learning institutions identified as having significant numbers of refugee students aged between 15 and 24 years will be targeted. The research package will be given to youth who express interest in participating in the project.

Additionally, groups and organizations having extensive contact with refugee populations will assist with the recruitment of initial participants who in turn will refer other potential participants to the study. A range of promotion strategies in multiple community settings will be used to ensure the sample is as representative as possible. The project will be widely promoted through multicultural service providers, refugee community leaders, schools, refugee community events, and ethnic community radio/media using brochures, flyers, letters, and a Call for Volunteers sheet.

Youth will be recruited for individual interviews through an Expression of Interest form they receive after completing the survey. Parents or caregivers of those participants who complete an interview will also be interviewed. Teachers will be recruited from among staff where youth participants are studying.

### Study Design and Data Collection

A survey of 600 refugee youth (with approximately 200 from each focus region) will form a major foundation of the study. A survey will be developed, and detailed pilot testing will be undertaken to ensure that the instrument is able to produce meaningful and robust results.

The qualitative component will purposively target 60 youth from the larger sample. These youths, their parents/caregivers, and their teachers will be individually interviewed. Interview questions will be constructed from survey data, investigating key challenges and successes at school, transitional issues, knowledge of the Australian education system including educational pathways, and family influences on education-based decision making.

Interviews and surveys will be completed at multiple venues at the convenience of participants. Venues will include the Multicultural Youth South Australia (MYSA) and Australian Migrant Resource Centre (AMRC) premises, interview or counseling rooms at other organizational premises, schools, participants’ homes, or public libraries.

### Selection and Training of Bilingual Workers

A group of bilingual youth workers will deliver the research package and administer the survey. This will enable enhanced communication so nothing is missed in interpretation. The bilingual youth workers will receive training and a comprehensive instruction manual providing background research information, promotional strategies, participant eligibility criteria, an explanation of the research questions, detailed instructions for their administration, and important ethical considerations.

### Translation of Study Materials

Study materials will be translated, as required, into the target languages by translators accredited by the National Accreditation Authority for Translators and Interpreters and back-translated by an independent bilingual professional from each of the target communities with knowledge of concepts. A panel of language and content experts will examine the adequacy of the original translation and will advise on use of correct terms to ensure translation conveys the meaning and the exact concepts are maintained. The survey will be pretested with three youth from each of the target communities, and revised if necessary before being administered to the target groups. The cover letter, information page, and consent form will also be recorded as audio (in the target languages) for parents/caregivers who are illiterate in their own language.

### Ethical Considerations

In line with our collaborative approach, a reference group consisting of ethnic community representatives and external senior researchers with transcultural research expertise will be established to ensure the research is carried out in partnership with the communities in a culturally appropriate manner and the results are relevant to the community. Bilingual youth workers will ensure clear understanding of the survey questions, and interpreters will be available at all times for youth or their families to clarify concepts or terms when necessary.

All researchers and bilingual youth workers who work with youth will have a National Criminal History Record Check and Screening Assessment, which is a requirement for people working with children in Australia. Individual contact details of participants will be recorded in a password-protected file kept separately from survey and interview data. Digital copies of the interview recordings will also be kept separately from de-identified transcriptions and translations.

**Figure 1:**
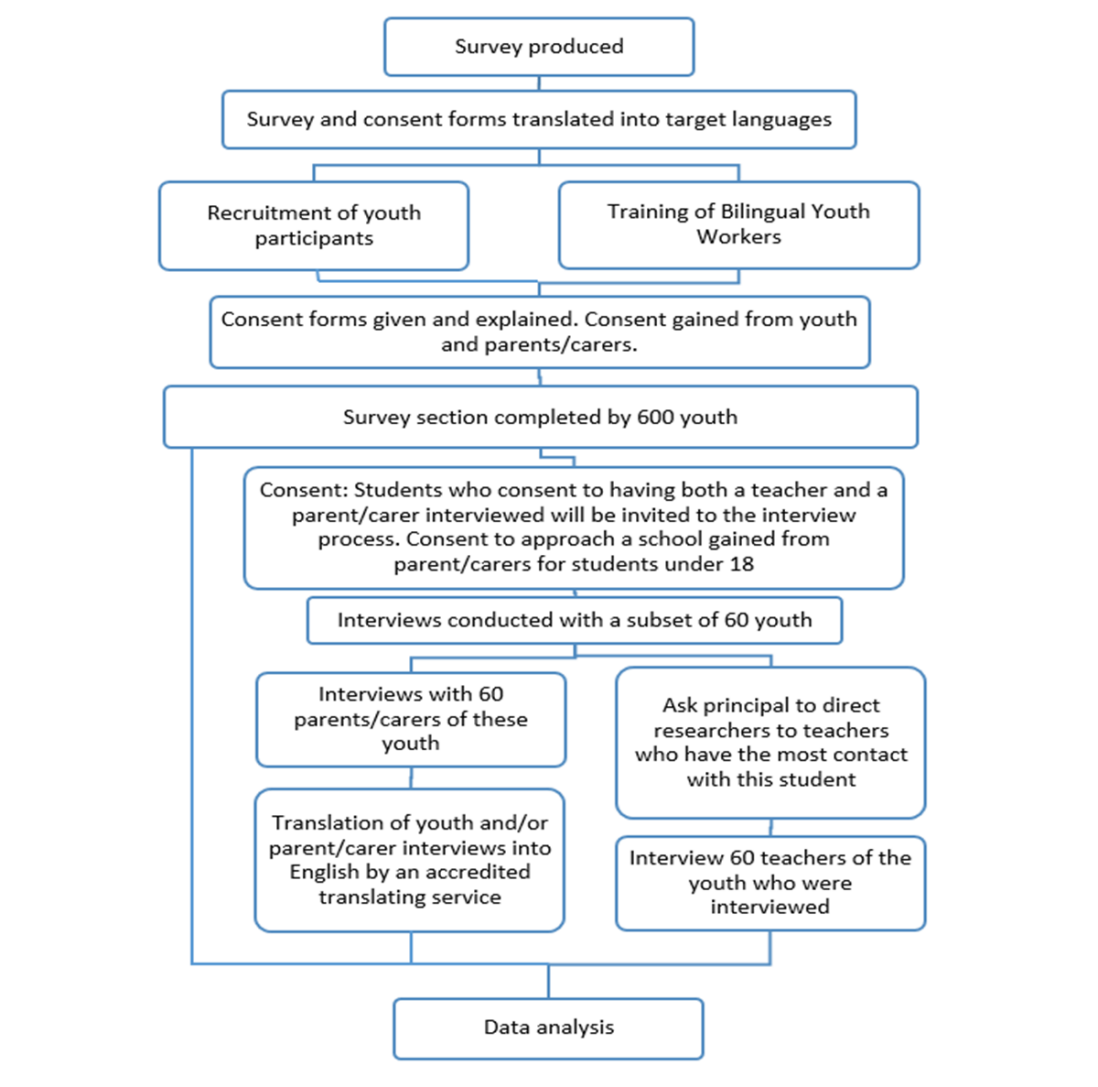
Study protocol.

Prior to any data collection, detailed information will be given to participants (and parents/caregivers for those aged under 18 years) and appropriate consent will be sought and gained for each stage of the study. Participants will be informed that they may withdraw at any time without any consequence and any related data will be excluded from the study. An option to be referred to appropriate counseling services will be offered to participants who may become distressed from the research experience. See Figure 1 for the study protocol.

## Results

### Data Collection

This study is due for completion by the end of 2019. It began in 2016, and data collection commenced in 2017. The quantitative survey component of the study currently includes 635 participants and was closed in March 2019. The remaining qualitative data collection is currently in progress, with an in-progress total of 93 interview participants including educators, youth, and family members of the youth. Preliminary analysis of quantitative data is currently underway and is expected to be reported in 2019-2020. Similarly, qualitative data will be analyzed once the data set is complete and is expected to be presented in 2019-2020.

### Statistical Analysis and Data Management

With respect to quantitative data analysis, standard statistical techniques will be used. The response rate will be presented followed by a comparison of the demographics of the respondents with that for refugee youth as a whole in South Australia. Descriptive statistics will be presented with counts and percentages for categorical variables and means and standard deviations (or nonparametric equivalents) for continuous measures. Confidence intervals will be provided for main results. Tables will be provided for cross-tabulations by gender, migration region, and gender by migration region. Chi-square tests will be undertaken for comparisons of categorical variables by gender and migration region and analysis of variance undertaken for continuous variables. Time since arrival in Australia will be used as a covariate in all analysis where this is appropriate. However, these analyses will be undertaken in the spirit of hypothesis generation rather than hypothesis testing and levels of significance interpreted in this light. Regression models may also be employed for exploratory analysis purposes.

With respect to qualitative data analysis, the interviews will be recorded as audio and transcribed verbatim [9]. The data will be prepared, organized, and analyzed using a thematic approach following the principles and procedures suggested by Krueger and Casey [39]. The data will be presented in the form of detailed description using case illustrations, paraphrases, and direct quotations from the identified themes.

## Discussion

### Summary

This project seeks to provide benefits for refugee youth during and after the research process. It is hoped that benefits will also accrue to the broader refugee community as a result of the researchers’ advocacy activities by disseminating the findings to policy makers, services planners, and others strategically placed to take action and make changes on behalf of refugee youth populations. In keeping with the advocacy interest in ensuring that the people affected by an issue are enabled to take action on their own behalf [35], the knowledge generated through the research will be made available to refugee communities, multicultural agencies, organizations, and youth advocacy bodies.

Building on earlier studies [1,6,12,40], this project incorporates new areas for investigation and involves collaboration between researchers from the University of South Australia, the University of Adelaide, and industry partners MYSA and AMRC, the two leading settlement agencies for refugee youth and families in South Australia.

The successful settlement of refugee youth as fully participating Australian citizens is desirable both for our society and for the individuals concerned as it directly affects their contribution to the nation at large [1,2,7]. Until recently, the literature on migration and education outcomes has failed to take account the experiences of refugees as distinct from those of other migrants [7,41]. Education policy makers have focused mainly on migrant and multicultural education. These exclusions from academic research and public policy provide a context for targeted policies and frameworks. Attention needs to be given to the education and training needs of refugee youth who are at high risk in a complex transition process [7,19,41].

The transition from school to employment is not always linear; rather, young people may take a pathway that leads to partial employment or further education before successfully embarking on a career path. Effective transitions require individual self-efficacy and motivation combined with resources such as family or social connections, educational qualifications, vocational training, and knowledge. A relative weakness in one of these areas can be made up for with a strength elsewhere, but a combination of poor resources and a lack of individual agency results in a likelihood of more negative transition outcomes [42]. Smooth transitions can therefore be facilitated by improving access to education and training and by building individuals’ belief in their skills, abilities, and capability to make informed choices regarding the future.

As people with a refugee background access and participate in education and employment, the likelihood of their successful integration into the larger community increases [20,43]. With effective delivery, education can not only support well-being and connection for young people [44] but it is also a means to employment through the provision of vocational skill development, support of young people’s maturity, and establishment of opportunities for professional intervention [21].

This research addresses an issue of significant economic and social concern. There is enormous potential for refugees’ economic, social, and civic contribution, and it is crucial that finely tuned policies work to realize that potential [1,12].

### Conclusion

This project will contribute new and unique insights to knowledge in relation to key factors influencing education and employment outcomes among refugee youth. It will bridge identified gaps in the knowledge of researchers and government policy makers who, as part of their focus on employment, are concerned to support research partnerships that will result in improved employment outcomes for disadvantaged groups such as refugees [1,7].

There are very few studies focusing on this key transitional phase between school and further education or employment, particularly with the inclusion of both generation 1 and generation 1.5 (youth born overseas who arrived in Australia as adolescents or children). This project will be the first of its kind undertaken in Australia and will contribute new insights resulting in significant improvements to the long-term employment opportunities of refugee youth. Factors that help and hinder young refugees in achieving their desired education, training, and employment goals will be identified. Findings will assist agencies and professionals within these sectors to identify and plan strategic support, and existing approaches will be assessed to further increase the capacity of refugee youth to achieve their desired employment goals. This research will enable effective planning for the needs of some of Australia’s most disadvantaged and marginalized young people, leading to a sustainable improvement in the education and employability of young refugees.

## Appendix

Multimedia Appendix 1 Demographic Profile of South Australian Refugee Youth Population 2019.

## Abbreviations

AMRC: Australian Migrant Resource Centre
MYSA: Multicultural Youth South Australia

## References

[1] Hugo G, Vas Dev S, Wall J, Young M, Sharma V, Parker K (2011). Economic, Social and Civic Contributions of First and Second Generation Humanitarian Entrants: Final Report prepared for Department Of Immigration And Citizenship [Thesis].

[2] Colic-Peisker V, McKay S (2009). The “visibly different” refugees in the Australian labor market: settlement policies and employment realities. Refugees, Recent Migrants and Employment Challenging Barriers and Exploring Pathways.

[3] Earnest J, Joyce A, de Mori G, Silvagni G (2010). Are universities responding to the needs of students from refugee backgrounds?. Aust J Educ.

[4] Cassity E (2007). Voices shaping education: young African refugees in Western Sydney high schools. Int Educ J Compar Perspect.

[5] Kalter F, Kogan I (2006). Ethnic inequalities at the transition from school to work in Belgium and Spain: discrimination or self-exclusion?. Res Soc Stratif Mobil.

[6] de Anstiss H, Ziaian T (2010). Mental health help-seeking and refugee adolescents: qualitative findings from a mixed-methods investigation. Aust Psychol.

[7] Taylor S, Sidhu R (2012). Supporting refugee students in schools: what constitutes inclusive education?. Int J Inclusive Educ.

[8] Miller E, Ziaian T, Esterman A (2017). Australian school practices and the education experiences of students with a refugee background: a review of the literature. Int J Inclusive Educ.

[9] Anisef P, Sweet R, Frempong G (2003). Labour market outcomes of immigrant and racial minority university graduates in Canada. Int Migrat Integrat.

[10] Stevenson J, Willott J (2007). The aspiration and access to higher education of teenage refugees in the UK. Compare J Compar Int Educ.

[11] (2010). Economic, civic and social contributions of refugees and humanitarian entrants—literature review.

[12] Hugo G (2013). The economic contribution of humanitarian settlers in Australia. Int Migr.

[13] (2011). Refugee status report: a report on how refugee children and young people in Victoria are faring.

[14] (2017). 2016 Census: Multicultural—Census reveals a fast changing, culturally diverse nation.

[15] (2009). Refugee and humanitarian issues: Australia’s response.

[16] Hugo G, McDougall K, Tan G, Feist H (2014). The CALD Youth Census Report 2014: the first australian census data analysis of young people from culturally and linguistically diverse backgrounds.

[17] Birman D, Trickett E (2016). Cultural transitions in first-generation immigrants: acculturation of Soviet Jewish refugee adolescents and parents. J Cross-Cultural Psychol.

[18] Brooker A, Lawrence J (2012). Educational and cultural challenges of bicultural adult immigrant and refugee students in Australia. Aust J Adult Learn.

[19] Kovacev L, Shute R (2016). Acculturation and social support in relation to psychosocial adjustment of adolescent refugees resettled in Australia. Int J Behav Devel.

[20] King S, Owens L, Askell-Williams H (2015). The schooling experiences of African youth from refugee backgrounds in South Australia: key findings and implications for educational practice. Transforming the Future of Learning with Educational Research.

[21] Hutchinson J, Kettlewell K (2015). Education to employment: complicated transitions in a changing world. Educ Res.

[22] Nicolai S, Triplehorn C (2003). Network Paper: the role of education in protecting children in conflict.

[23] Datnow A, Hubbard L, Mehan H (2002). Extending Educational Reform: From One School to Many.

[24] Brown T, Rodríguez L (2009). School and the co‐construction of dropout. Int J Qual Stud Educ.

[25] Minza W (2012). Young migrants and education-to-work transitions in Pontianak, West Kalimantan. Asia Pac J Anthropol.

[26] Nielsen HS, Rosholm M, Smith N, Husted L (2003). The school-to-work transition of 2nd generation immigrants in Denmark. J Popul Econ.

[27] Correa-Velez I, Onsando G (2009). Educational and occupational outcomes amongst African men from refugee backgrounds living in urban and Regional Southeast Queensland. Australasian Rev African Stud.

[28] Watkins PG, Razee H, Richters J (2012). “I'm telling you ... the language barrier is the most, the biggest challenge”: barriers to education among Karen refugee women in Australia. Aust J Educ.

[29] Earnest J, Mansi R, Bayati S, Earnest J, Thompson S (2015). Resettlement experiences and resilience in refugee youth in Perth, Western Australia. BMC Res Notes.

[30] Rah Y, Choi S, Nguyễn T (2009). Building bridges between refugee parents and schools. Int J Leadership Educ.

[31] Stevens C (2016). The school to work transition: young Cambodians in South Australia. Aust NZ J Sociol.

[32] Wilkinson L (2008). Labor market transitions of immigrant-born, refugee-born, and Canadian-born youth. Can Rev Sociol.

[33] Uptin J, Wright J, Harwood V (2012). 'It felt like I was a black dot on white paper': examining young former refugees' experience of entering Australian high schools. Aust Educ Res.

[34] Portes A, Fernández-Kelly P, Haller W (2005). Segmented assimilation on the ground: the new second generation in early adulthood. Ethn Racial Stud.

[35] Mertens D, Tashakkori A, Teddlie C (2003). Mixed methods and the politics of human research: the transformative-emancipatory perspective. Handbook of Mixed Methods in Social & Behavioral Research.

[36] Creswell J (2003). Research Design: Qualitative, Quantitative, and Mixed Methods Approaches. 2nd Edition.

[37] Bhugra D (2004). Migration, distress and cultural identity. Br Med Bull.

[38] Gilner J, Morgan G (2000). Research Methods in Applied Settings: An Integrated Approach to Design and Analysis.

[39] Krueger R, Casey M (2000). Focus Groups: A Practical Guide for Applied Research. 4th Edition.

[40] Ziaian T, de Anstiss H, Antoniou G, Sawyer M, Baghurst P (2011). Depressive symptomatology and service utilisation among refugee children and adolescents living in South Australia. Child Adolesc Ment Health.

[41] Matthews J (2008). Schooling and settlement: refugee education in Australia. Int Stud Sociol Educ.

[42] Furlong A, Cartmel F, Biggart A, Sweeting H, West P (2003). Youth transitions: patterns of vulnerability and processes of social inclusion.

[43] Khawaja N, Hebbani A (2017). Does employment status vary by demographics? An exploratory study of former refugees resettled in Australia. Aust Soc Work.

[44] Ziaian T, de Anstiss H, Puvimanasinghe T, Miller E (2017). Refugee students’ psychological wellbeing and experiences in the Australian education system: a mixed-methods investigation. Aust Psychol.

