# Supplementary material for: Refugee Youth and Transition to Further Education, Training, and Employment in Australia: Protocol for a Mixed Methods Study
Source: JMIR Res Protoc. 2019 Jul 31;8(7):e12632. doi: 10.2196/12632 (PMC6693298; doi:10.2196/12632)
Supplement: Multimedia Appendix 1 [file resprot_v8i7e12632_app1.pdf]

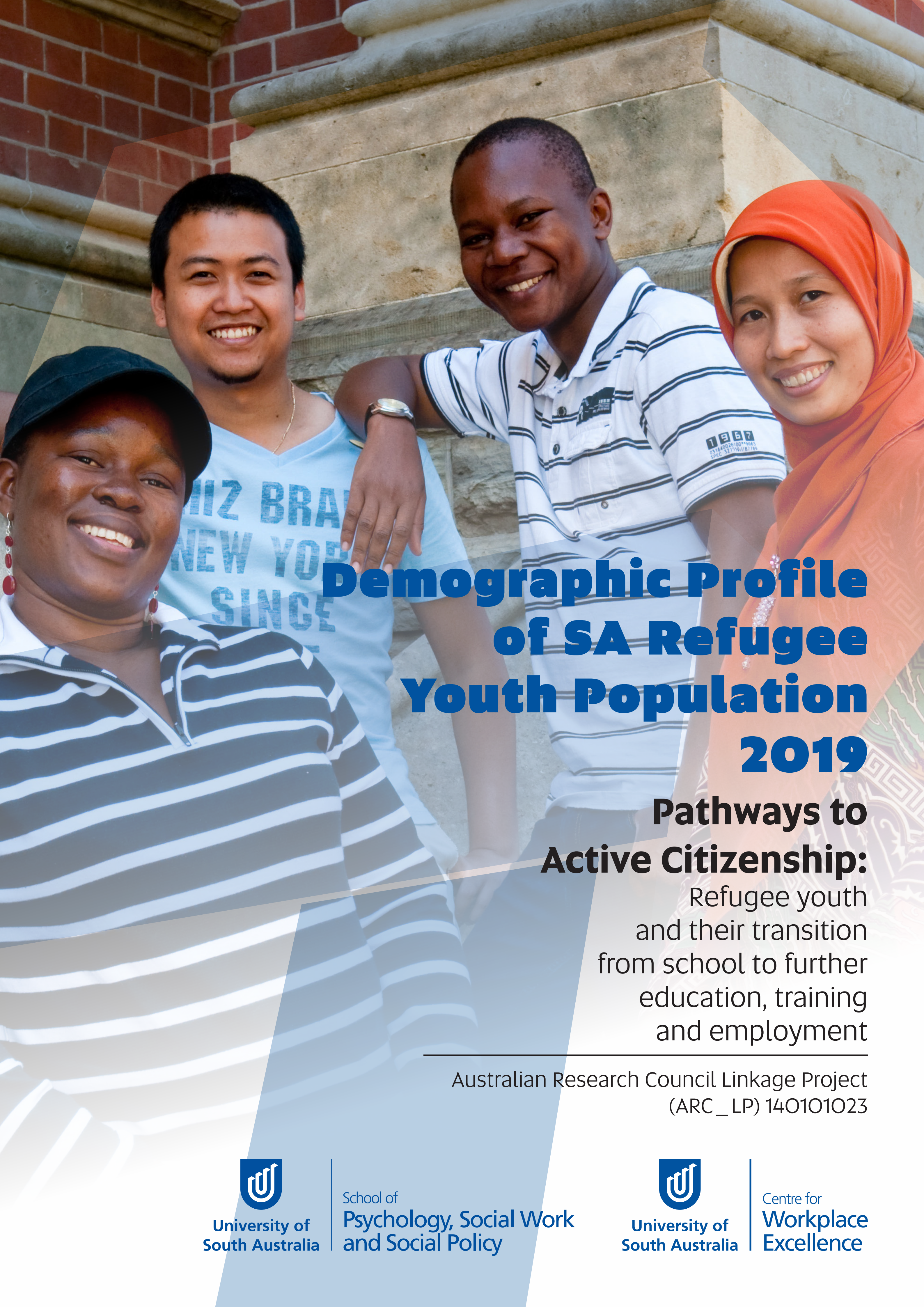

Demographic Profile of SA Refugee Youth Population 2019

Pathways to Active Citizenship:

Refugee youth and their transition from school to further education, training and employment

Australian Research Council Linkage Project (ARC_LP) 140101023

University of South Australia
School of Psychology, Social Work and Social Policy

University of South Australia
Centre for Workplace Excellence

Copyright University of South Australia 2019. This work is copyright. Apart from any use as permitted under the Copyright Act 1968, no part may be reproduced by any means or process without prior written permission of the copyright owners. Published by University of South Australia (School of Psychology, Social Work & Social Policy).
DOI: https://doi.org/10.25954/5c90261b25e47 ISBN: 978-0-646-80424-8

University of
South Australia

Pathways to Active Citizenship: Refugee youth and their transition from school to further education, training and employment

Australian Research Council Linkage Project (ARC_LP) 140101023

Demographic Profile of SA Refugee Youth Population

2019

Centre for Workplace Excellence, Business School

School of Psychology, Social Work and Social Policy

University of South Australia

CENTRE FOR WORKPLACE EXCELLENCE | University of South Australia | Business School

THE UNIVERSITY OF ADELAIDE AUSTRALIA
AUSTRALIAN POPULATION AND MIGRATION RESEARCH CENTRE

MYSA Multicultural Youth SA Inc.

AMRC Australian Migrant Resource Centre

Report Authors

Associate Professor Tahereh Ziaian
University of South Australia.

Dr Helen Barrie
Research Fellow
University of Adelaide

Dr Teresa Puvimanasinghe
Research Associate
University of South Australia

Ms Emily Miller
Research Assistant
University of South Australia

Professor Maureen Dollard
Co-Director, Centre for Workplace Excellence
University of South Australia

Professor Adrian Esterman
Chair of Biostatistics
University of South Australia

Dr Helena de Anstiss
Senior Lecturer
University of South Australia

Dr Ali Afsharian
Research Assistant
University of South Australia

Partner Organisations

Multicultural Youth, South Australia (MYSA)
Ms Tamara Stewart-Jones, CEO
Ms Susie Wilkes, Project Coordinator

Australian Migrant Resource Centre (AMRC)
Ms Eugenia Tsoulis, OAM, CEO
Ms Cynthia Caird, Manager
Ms Michelle Dieu, Manager

Table of Contents

List of Figures iv
List of Tables iv
FOREWORD vii
EXECUTIVE SUMMARY viii
Section A: Introduction to the research viii
Section B: Refugee Youth 15-24 years – Australia and South Australia: Census Data viii
Section C: Refugee Youth 15-24 years – South Australia: Survey Data x
A. Introduction 1
1. Background 1
2. Aims and Objectives 3
3. Selection Criteria 3
B. Refugee Youth 15-24 years – Australia and South Australia: Census Data 7
1. Refugee Youth by Region in Australia and South Australia 7
2. South Australia: Analysis by Regions over Time 10
3. Profile of Youth 15-24 years by Refugee Birthplace Groups for South Australia (Arrived 2012–2016) 13
3.1. Nominated Refugee Countries in the Africa Region 13
3.1.1 Sudan 13
3.1.2 South Sudan 15
3.1.3 Republic of Congo 16
3.1.4 Democratic Republic of Congo 17
3.1.6 Ethiopia 19
3.1.7 Liberia 20
3.1.7 Somalia 21
3.1.8 Overview of Refugee Youth Arriving between 2012 and 2016 in the African Region 22
3.2 Nominated Refugee Countries in the Middle East Region 23
3.2.1 Afghanistan 23
3.2.2 Syria 25
3.2.3 Iran 26
3.2.4 Iraq 28
3.2.5 Overview of Refugee Youth Arriving between 2012 and 2016, Middle East Region 29
3.3 Nominated Refugee Countries in the South Asia Region 30
3.3.1 Myanmar 30
3.3.2 Bhutan 31
3.3.3 Nepal 32
3.3.4 Pakistan 33
3.3.5 Overview of Refugee Youth Arriving between 2012 and 2016, South Asia Region 35
4. Key points from all birthplace groups 36
C. Refugee Youth 15-24 years –South Australia: Survey Data 37

1. Cultural and Demographic Background of Participants........38
2. Participant's Life Course Experiences before or immediately after Arriving in Australia.....42
References........44

List of Figures

Figure 1: Refugee Youth (15 to 24 Years) 2006, 2011 and 2016 for Australia by Birthplace Country and Region....8
Figure 2: Refugee Youth (15 to 24 Years) 2006, 2011 and 2016 for South Australia by Birthplace Country and Region........8
Figure 3: Percentage of Total Refugee Youth (15 to 24 years) by Region for Australia, 2006, 2011 and 2016........9
Figure 4: Percentage of Total Refugee Youth (15 to 24 years) by Region for South Australia, 2006, 2011 and 2016........9
Figure 5: Participants' Age........39
Figure 6: Length of Residency in Australia........40

List of Tables

Table 1 Comparison of Youth Aged 15-24 years in 2016........7
Table 2 Comparison of Refugee Youth (15 to 24 Years) 2006, 2011 and 2016 for Australia and South Australia by Birthplace Region and Country........7
Table 3: Youth 15-24 years from Refugee Countries of Birth by Regions in South Australia, 2006.....10
Table 4: Youth 15-24 years from Refugee Countries of Birth by Regions in South Australia, 2011.....11
Table 5: Youth 15-24 years from Refugee Countries of Birth by Regions in South Australia, 2016.....12
Table 6: Overview of the Sudanese Population, South Australia........13
Table 7: Employment Status, Newly Arrived (2012-2016) Sudanese Youth, South Australia........14
Table 8: Education Participation, Newly Arrived (2012-2016) Sudanese Youth, South Australia........14
Table 9: Type of Educational Institution Attending, Newly Arrived (2012-2016) Sudanese Youth, South Australia........14
Table 10: Highest Level of Schooling Completed in 2016, Newly Arrived (2012-2016) Sudanese Youth, South Australia........14
Table 11: Overview of the South Sudanese Population, South Australia........15
Table 12: Employment Status, Newly Arrived (2012-2016) South Sudanese Youth, South Australia..15
Table 13: Education Participation, Newly Arrived (2012-2016) South Sudanese Youth, South Australia........15
Table 14: Type of Educational Institution Attending, Newly Arrived (2012-2016) South Sudanese Youth, South Australia........16
Table 15: Highest Level of Schooling Completed in 2016, Newly Arrived (2012-2016) South Sudanese Youth, South Australia........16
Table 16: Overview of the Republic of Congo Population, South Australia........16
Table 17: Overview of the Democratic Republic of Congo Population, South Australia........17
Table 18: Place of Residence for Newly Arrived (2012-2016) Congolese (DRC) Youth, South Australia........17
Table 19: Education Participation, Newly Arrived (2012-2016) Congolese (DRC) Youth, South Australia........18
Table 20: Type of Educational Institution Attending, Newly Arrived (2012-2016) Congolese (DRC) Youth, South Australia........18
Table 21: Highest Level of Schooling Completed in 2016, Newly Arrived (2012-2016) Congolese (DRC) Youth, South Australia........18

Table 22: Overview of the Ethiopian Population, South Australia 2016 19
Table 23: Employment Status, Newly Arrived (2012-2016) Ethiopian Youth, South Australia 19
Table 24: Education Participation, Newly Arrived (2012-2016) Ethiopian Youth, South Australia 19
Table 25: Type of Educational Institution Attending, Newly Arrived (2012-2016) Ethiopian Youth, South Australia 20
Table 26: Highest Year of Schooling Completed in 2016, Newly Arrived (2012-2016) Ethiopian Youth, South Australia 20
Table 27: Overview of the Liberian Population, South Australia 2016 20
Table 28: Overview of the Somalian Population, South Australia 2016 21
Table 29: Overview of Population for Refugee Nominated Countries across the Africa Region for South Australia, 2016 22
Table 30: Overview of the Afghani Population, South Australia 2016 23
Table 31: Place of Residence for Newly Arrived (2012-2016) Afghani Youth, South Australia 23
Table 32: Citizenship, Newly Arrived (2012-2016) Afghani Youth, South Australia 24
Table 33: Employment Status, Newly Arrived (2012-2016) Afghani Youth, South Australia 24
Table 34: Education Participation, Newly Arrived (2012-2016) Afghani Youth, South Australia 24
Table 35: Type of Educational Institution Attending, Newly Arrived (2012-2016) Afghani Youth, South Australia 24
Table 36: Highest Year of Schooling Completed as of 2016, Newly Arrived (2012-2016) Afghani Youth, South Australia 25
Table 37: Overview of the Syrian Population, South Australia 2016 25
Table 38: Education Participation, Newly Arrived (2012-2016) Syrian Youth, South Australia 25
Table 39: Type of Educational Institution Attending, Newly Arrived (2012-2016) Syrian Youth, South Australia 26
Table 40: Highest Year of Schooling Completed, Newly Arrived (2012-2016) Syrian Youth, South Australia 26
Table 41: Overview of the Iranian Population, South Australia 2016 26
Table 42: Place of Residence for Newly Arrived (2012-2016) Iranian Youth, South Australia 27
Table 43: Citizenship, Newly Arrived (2012-2016) Iranian Youth, South Australia 27
Table 44: Employment Status, Newly Arrived (2012-2016) Iranian Youth, South Australia 27
Table 45: Education Participation, Newly Arrived (2012-2016) Iranian Youth, South Australia 27
Table 46: Type of Educational Institution Attending, Newly Arrived (2012-2016) Iranian Youth, South Australia 28
Table 47: Highest Year of Schooling Completed, Newly Arrived (2012-2016) Iranian Youth, South Australia 28
Table 48: Overview of the Iraqi Population, South Australia 2016 28
Table 49: Education Participation, Newly Arrived (2012-2016) Iraqi Youth, South Australia 29
Table 50: Overview of Population for Refugee Nominated Countries across the Middle East Region for South Australia, 2016 29
Table 51: Overview of the Myanmarese Population, South Australia 2016 30
Table 52: Place of Residence for Newly Arrived (2012-2016) Myanmarese Youth, South Australia ... 31
Table 53: Employment Status, Newly Arrived (2012-2016) Myanmarese Youth, South Australia 31
Table 54: Education Participation, Newly Arrived (2012-2016) Myanmarese Youth, South Australia 31
Table 55: Type of Educational Institution Attending, Newly Arrived (2012-2016) Myanmarese Youth, South Australia 31
Table 56: Overview of the Bhutanese Population, South Australia 2016 32
Table 57: Overview of the Nepalese Population, South Australia 2016 32
Table 58: Employment Status, Newly Arrived (2012-2016) Nepalese Youth, South Australia 33

Table 59: Education Participation, Newly Arrived (2012-2016) Nepalese Youth, South Australia 33
Table 60: Type of Educational Institution Attending, Newly Arrived (2012-2016) Nepalese Youth, South Australia 33
Table 61: Highest Year of Schooling Completed, Newly Arrived (2012-2016) Nepalese Youth, South Australia 33
Table 62: Overview of the Pakistani Population, South Australia 2016 34
Table 63: Place of Residence for Newly Arrived (2012-2016) Pakistani Youth, South Australia 34
Table 64: Employment Status, Newly Arrived (2012-2016) Pakistani Youth, South Australia 34
Table 65: Education Participation, Newly Arrived (2012-2016) Pakistani Youth, South Australia 34
Table 66: Type of Educational Institution Attending Newly Arrived (2012-2016) Pakistani Youth, South Australia 35
Table 67: Overview of Population for Refugee Nominated Countries across the South Asia Region for South Australia, 2016 35
Table 68: Cultural / Ethnic Background of Survey Participants 38
Table 69: Participants' Migration by Region 38
Table 70: Participants' Gender 39
Table 71: Participants' Age 39
Table 72: Participants' Age: Over vs. Under 18. 39
Table 73: Length of Residency in Australia 40
Table 74: Participants' Employment Status 41
Table 75: Participants' Main Activity 41
Table 76: Participants' Refugee Camp Experience 42
Table 77: Participants' Detention Camp Experience 42
Table 78: Participants' Previous Trauma Experiences 43

FOREWORD

It is with great pleasure that I recommend this research report, as a result of the work by the "Pathways to Active Citizenship" research team and published by the University of South Australia, to all those who work with new immigrants from refugee backgrounds and their communities.

The successful settlement and integration of migrants and refugees is a critical issue for these individuals, their communities, and society as a whole. Our national data indicate that almost two thirds of recent arrivals of people from refugee background have not yet reached the age of 30. These young Australians have great potential to thrive if appropriate opportunities are available to them.

The report presents strong evidence of our contemporary diverse population, showcasing that alongside established communities a new and incredible pattern of cultural and linguistic diversity in Australia is emerging. It is important that we equip young Australians of all backgrounds with the training, education, encouragement and support they need to fulfil their aspirations.

This report is a useful tool which provides an evidence base for decision making in education, training and employment policy and practices. I have no doubt it will become a standard reference for educators, employers and practitioners across a diverse range of disciplines, not only in Australia but in other resettlement countries as well.

His Excellency the Honourable Hieu Van Le AC

Governor of South Australia

EXECUTIVE SUMMARY

Australia is a multicultural society made up of individuals with a wide range of cultural histories and identities. Australian people's variation in cultural and linguistic heritage ranges from Aboriginal Australians with a link to country that has been continuous for thousands of years, to people who have migrated to Australia very recently. Australia's history of immigration over the last two centuries has included people arriving from all over the globe and currently nearly half of all Australian people were either born overseas or have one parent who was born overseas (Australian Bureau of Statistics (ABS), 2016).

A portion of migration to Australia is made up of people who resettle via the humanitarian scheme. This scheme provides resettlement for people who are fleeing persecution as refugees. Almost two thirds of recent arrivals of people from refugee backgrounds were under the age of 30 (Department of Home Affairs (DOHA), 2018). A majority of these young people access education in Australia and actively work towards their aspirations. These young people may encounter some challenges that Australian born students or those with migrant backgrounds do not, but overall their educational performance eventually compares well with these peers (Graham, Minhas, & Paxton, 2016).

The data presented here were collected as part of an Australian Research Council funded study investigating educational outcomes and pathways through school and on to further education, training or employment for young South Australians, aged 15-24, from refugee backgrounds. Researchers from the University of South Australia and the University of Adelaide conducted the research in collaboration with Industry Partner Multicultural Youth South Australia (MYSA) and Research Partner Australian Migrant Resource Centre (AMRC). The research began in 2015 and is due for completion in 2019.

This project aimed to find out about the ways in which young people successfully engaged in education and what helped them to access pathways to further education, training or employment. As part of this investigation, Australian Census data from 2006, 2011 and 2016 were collected and are presented in this report. In addition, this report includes some of the sociodemographic data collected during the survey phase of this project.

Section A: Introduction to the research

This section of the report describes the research process. This research focused on young people with refugee backgrounds from three migration regions: Africa, the Middle East and South Asia. These regions were chosen in response to the major migration patterns in the decade prior to project commencement. Although the ratio of people from particular countries of origin altered over time, these three regions remained dominant. Quantitative and qualitative data were collected from young people, their families and their teachers or educators. These data and their analyses will be presented in detail elsewhere in the form of journal publications.

Section B: Refugee Youth 15-24 years – Australia and South Australia: Census Data

The primary section of this report presents Australian Bureau of Statistics Census of Population and Housing data pertaining to Australian youth aged 15-24, with a focus on South Australian youth, with refugee backgrounds in 13 countries from within the three migration regions, namely:

- the seven African nations of the Democratic Republic of Congo, Ethiopia, Liberia, the Republic of Congo, Somalia, South Sudan, and Sudan;
- the four Middle Eastern nations of Afghanistan, Iran, Iraq and Syria; and,
- the four South Asian nations of Bhutan, Myanmar, Nepal and Pakistan.

Census data from 2006, 2011 and 2016 were collected, focusing on these 13 key countries. Trends and variations across Australia and South Australia are reported in subsection 1, followed by a focused investigation of South Australian trends in subsection 2. The report provides detailed South Australian data for each of the countries identified, with a focus in subsection 3 on the changes recorded in the most recent Census of 2016.

Subsection 1: Refugee youth by region in Australia and South Australia

The first sub-section examines Census data over time for all youth aged 15 to 24 years, comparing figures for Australia and South Australia over three Census periods 2006, 2011 and 2016. In 2016, youth from the nominated refugee countries of birth, aged 15 to 24 represented only 2.4% of all youth in Australia; whereas for South Australia youth from these countries of birth represented a slightly higher proportion of the youth population at 2.7%. South Australia showed a similar pattern of change to Australia over the past decade, with steadying numbers of refugee youth from the African countries, slight increases from the Middle East but the largest increases coming from the South East Asia region. In fact, the numbers for South Asia rose from 493 youth in 2006 to almost 3,500 in 2016, an increase of 686%.

Subsection 2: South Australia: Analysis by Regions over Time

The Census data presented in this section include distributions of youth across the different South Australian regions. Settlement patterns across South Australia were recorded as a rise in regional settlement (from 50 youths from our target population in 2006 to 232 in 2016). The regional youth population was mainly centred on the Limestone Coast/South East region. This was to be expected as this is the only planned settlement site for refugee families in regional SA. In 2011 and 2016 the northern and western regions of Adelaide (particularly the northern areas) remained the most highly populated by refugee youth, and most increases were seen in these regions over this period, while the southern and eastern regions of Adelaide remained steady.

Subsection 3: Profile of Youth 15-24 years by Refugee Birthplace Groups for South Australia (Arrived 2012–2016)

This section explores each of the nominated refugee birthplace groups in more detail using the 2016 ABS Census of Population and Housing data. Data is presented on English proficiency, language spoken at home, citizenship, and employment and student status for each birthplace group.

The seven nominated African countries represented just over 20% of South Australia's youth population which was a decrease from 43.6% in 2006. Although the percentage dropped, the overall numbers of youth from African backgrounds in the nominated countries increased at both the Australian level (from 7,504 in 2006 to 11,224 in 2016) and the South Australian level (from 752 in 2006 to 1,106 in 2016). Youth aged 15-24 years comprised 24.9% of the total population, however they made up 31.8% of all arrivals during the five years preceding the 2016 Census.

The four nominated Middle Eastern countries made up 42.3% of all refugee youth in Australia and 51.6% in South Australia at the time of the 2016 Census. Nationally there was an overall drop of 9.7%

in the percentage of youth from the nominated countries between the 2006 and 2016 Census, however in South Australia there was a slight rise of 3.1% due predominantly to the increased number of youth from Afghanistan settling in the state. For Australia the population numbers for youth aged 15 to 24 years almost doubled, from 13,847 in 2006 to 27,429 in 2016. For South Australia this increase saw the youth population for this region more than triple, from 836 in 2006 to 2,828 in 2016.

The four nominated South Asian countries made up 34.2% of all refugee youth in Australia and 28.5% in South Australia. By 2016, this group were 57.9% of the national refugee youth population but had also increased to form 61.6% of South Australia's refugee youth population aged 15 to 24 years. This represented an increase of more than seven times the 2006 population for this region, from 493 in 2006 to 3,381 in 2016.

Section C: Refugee Youth 15-24 years – South Australia: Survey Data

This section of the report details a selection of the research project's Primary data. Quantitative data collection was conducted between 2016 and 2018 via a detailed survey of 630 young people from refugee backgrounds currently living in South Australia. This report includes some of the sociodemographic data collected in this large-scale survey, including self-identified ethnic background, gender, age, previous experiences during the refugee journey, and current education and employment status.

Qualitative data collected via individual interviews with a smaller set of youth, their families and their teachers provided nuanced data relating to education and employment pathways and outcomes for these young people. Further information of the findings from the primary data collection and analysis conducted during this research project will be made available from 2019 via academic publications (Tahereh Ziaian et al., 2018).

The primary aim of this research was to provide an evidence base for decision making in education, training and employment policy and practice. The research presented in this report provides a strong underpinning for understanding of current socio-demographic factors relating to young people from refugee backgrounds in Australia and South Australia.

A. Introduction

1. Background

Australia is a multicultural society and many residents are first, second or third generation migrants, reflecting active government immigration and refugee resettlement programs throughout the nineteenth, twentieth and twenty first centuries (Hugo, 2010). Nearly half (49%) of all Australians were either born overseas or had at least one parent who was born overseas (Australian Bureau of Statistics (ABS), 2016). In 2016-2017, Australia accepted approximately 20,257 humanitarian entrants (refugees) including 13,739 displaced persons from conflict zones in Syria and Iraq (Department of Home Affairs (DOHA), 2018). Almost 58% of Australia's humanitarian intake were under 30 years of age (DOHA, 2018).

In 2015, the University of South Australia received an ARC Linkage Grant (LP140101023) for the 'Pathways to Active Citizenship Project'—to identify facilitators and barriers faced by refugee youth age 15-24 years, as they transition from school to further education, training and employment, and to investigate their access of available support systems and their awareness of educational and employment pathways. This project anticipated to provide vital information for the development of appropriate policies, services and support systems to improve educational and employment outcomes for refugee youth in Australia. The project has been in progress since 27 June 2015 and the current completion date is 30 June 2019.

Rationale. The successful settlement of refugee youth as fully participating Australian citizens is desirable for the individuals concerned, their families and communities, as well as for the wider Australian society because it directly affects their contribution to the nation at large (Cassity, 2013; Colic-Peisker, 2009; Hugo et al., 2011; Refugee Council of Australia, 2010). Their ability to engage with the labour market is an essential component of successful settlement and integration (Department of Education and Early Childhood Development, 2011; Hugo et al., 2011)

There is ample evidence that in the long-term people from refugee backgrounds do achieve positive employment outcomes (Refugee Council of Australia, 2010). There is also evidence that with time, the educational performance of refugee background youth becomes comparable to their non-refugee counterparts (Graham et al., 2016). However, the experiences of recently arrived refugee youth are significantly different from those who arrived during earlier time periods and who have been long-term residents of the host country (Berry, 1997). They face a different and unique set of challenges that place them at increased risk of poor educational outcomes and long-term unemployment compared with youth in the general population (Birman & Trickett, 2001; Taylor & Sidhu, 2012).

Although a large proportion of refugee background Australian youth receive their education in Australia (Hugo et al., 2011), they are at increased risk of poor education and employment outcomes (Cassity, 2013; Colic-Peisker, 2009). The challenges they encounter may include: limited English language skills; limited or interrupted former education; difficulty navigating the education, training, and employment systems; limited parental involvement in and support for their education; low teacher expectations; and, racism and discrimination (de Anstiss, Ziaian, Procter, Warland, & Baghurst, 2009; Graham et al., 2016; Hatoss, O'Neill, & Eacersall, 2012; Hugo et al., 2011; E. Miller, Ziaian, & Esterman, (2017); J. Miller, Mitchell, & Brown, 2005; Renzaho, McCabe, & Sainsbury, 2011;

Taylor & Sidhu, 2012). Pre-migration traumatic experiences fleeing war and persecution as well as post-migration distress such as fragmented family structures, dysfunctional family dynamics, disrupted community and social networks, poverty and intergenerational conflicts with parents and caregivers also adversely impact their education and employment outcomes (Dandy et al., 2017).

Refugee background youth must locate and connect themselves within a new cultural environment as well as try to find a sense of security within fractured families and communities (Cassity, 2007, 2013). The term 'transition' is particularly appropriate to describe the initial phase, post-migration, in the lives of refugee background youth, because it usually involves physical relocation from their country of origin to Australia (sometimes through other transit countries); transfer from a home language to English; adaptation to minority group status; adaptation to the Australian education system; transition from New Arrivals Program (NAP) or Intensive English Language Program (IELP) schools to mainstream schools; and transition from full-time education to the world of work (Dandy et al., 2017).

Many young refugees arrive in Australia at an age when they do not have sufficient time to learn English and adjust to the education system before the lead up to Year 12. English language and cultural barriers place them on an unequal footing with their Australian-born peers. Often there is no additional language or academic support at home because parents do not have English language skills to assist them. Adding further pressure is the unrealistic expectations that many parents place on them to enter highly competitive professions such as medicine, engineering, and dentistry.

High parental expectations are often tied to parents' sense of resignation about their own futures. Most parents suffer a loss of role and status with migration (Minza, 2012). Many are living on welfare payments or are working in unskilled, low-paid, and low-status jobs, which for the more educated and affluent represent a marked change from their pre-migration circumstances. Many young people feel their parents do not understand how difficult it is to excel in a new and unfamiliar education and employment environment (Dandy et al., 2017; de Anstiss et al., 2009).

Parents in general, lack knowledge of the career possibilities and directions available in Australia due to low involvement in educational and vocational guidance programs. English language difficulties and limited environmental mastery prevent many from attending parent interviews and other school activities designed to support children in their studies (Rah, Choi, & Nguyễn, 2009). Despite refugee-background youth indicating better than expected mental health (i.e. positive deviance) and comparable educational outcomes to that of peers (Graham et al., 2016; T. Ziaian, de Anstiss, Antoniou, Baghurst, & Sawyer, 2012, 2013), the numerous challenges they have encountered both pre- and post-migration, do place a disproportionate burden on their mental health and overall well-being. There is emerging evidence that refugee background youth are not accessing support services which could increase their prospects for successful integration as well as improve education and employment opportunities (Kovacev & Shute, 2004; Refugee Council of Australia, 2010).

Until recently, the literature on migration and education outcomes did not focus on the unique needs of refugee youth as distinct from those of other migrants (Matthews, 2008; Taylor & Sidhu, 2012). Their concerns were also ignored by educators and policymakers who mainly focused on migrant and multicultural education (Taylor & Sidhu, 2012). These exclusions from academic research and public policy provide a context for the lack of targeted policies and frameworks. Therefore, there is an urgent need for attention to be given to the education and training needs of

refugee youth who are at risk of being ‘left behind’ in a complex transition process (Kovacev & Shute, 2004; Matthew, 2008; Refugee Council of Australia, 2010; Taylor & Sidhu, 2012).

We anticipate that the present research project will contribute new and unique insights to knowledge in relation to key factors influencing further education and employment outcomes among refugee background youth. We intend that the study will bridge identified gaps in the knowledge of researchers and government policymakers and improve employment outcomes for disadvantaged groups such as people from refugee backgrounds (Hugo et al., 2011; Refugee Council of Australia, 2010; Taylor & Sidhu, 2012).

2. Aims and Objectives

Primary aim. The primary aim of the *“Pathways to Active Citizenship”* research project is to investigate education and employment outcomes among refugee background youth aged 15-24 years, in South Australia with a view to influencing education, training and employment policy and practice.

Secondary aims. The secondary aims of the project are to:

- Identify facilitators and barriers to successful transition from school into further education and employment;
- Map out the support systems accessed by youth who are experiencing education and employment-related difficulties; and
- Investigate the extent of youth and family awareness of available education, training and employment pathways.

The research is expected to lead to a sustainable improvement in the employability of refugee background youth by providing policy analysts and decision makers in the education, training, and employment sectors with information about a high profile yet under-researched population group. South Australia provides a broadly representative sample of refugee background youth since it takes a disproportionate share of refugee / humanitarian settlers (Hugo et al., 2011). Basing the study in South Australia will allow considerable depth to investigate state and local as well as federal influences, with the findings being of direct relevance to other States and Territories.

Better quality data collection is essential to ascertain the problems encountered by refugee background youth and to provide adequate support to them. Quality data collection will enhance our understanding of the importance of a specialist or targeted approach in mainstream youth programs. This report contributes to that baseline knowledge about refugee background youth in Australia.

3. Selection Criteria

Youth: The first selection criterion was that participating youth were between the ages of 15-24 years and engaged in some form of study at the time of participation. Project participation was restricted to youth who had migrated, or whose parents or caregivers had migrated between 12 months and 10 years[1] prior to the time of participation.

[1] The length of residency in Australia selection criterion was later extended to 15 years (Table 73).

The National Strategy for Young Australians (Australian Government, 2010) defines the term 'youth' as applicable to those between 12-24 years of age, with their economic productivity measured from 15 years onwards, in two age groups (15-19 and 20-24 years). Because the primary aim of the research project is to investigate education and employment outcomes among refugee background youth, the age range of 15-24 years was selected.

Length of residency in Australia: The second project criterion pertained to participants' length of residency in Australia. At project inception, this criterion was restricted to 1-10 years because the initial and middle stages of migration and resettlement present particular adjustment challenges, which are the focus of the study. Participants with less than one year of residency in Australia were excluded because the initial months in the resettlement country is especially stressful for newcomers, busy with settling into a new society, learning a new language, finding accommodation and familiarising themselves with basic systems. Therefore, newcomers with less than 1 year in Australia were deemed less suitable to participate in the research project.

Additionally, the selection criterion of 1-10 years ensured that almost all research participants belonged to Generation 1 or Generation 1.5 (i.e. those born overseas and arrived in Australia as adolescents or children). These youth were selected mainly because of their unique experiences and circumstances compared to that of their parents and Australian born siblings. That is, unlike their Australian born siblings, these youth are likely to be less proficient in English and to have experienced disruptions to their education due to reasons such as war, persecution, poverty, and refugee camp existence. They are also most likely to encounter difficulties navigating the local education system and schooling environment as well as transitioning from school to higher education, training and employment (Hugo, McDougall, Tan, & Feist, 2014). We also envisaged that refugee background youth belonging to General 1 or 1.5, would not have a strong foundation in the cultural, social and religious norms of their home country, unlike their parents' generation who arrived in Australia as adults. Hence, their identities would become split between the home and host countries, resulting in considerable identity confusion (Berry, 2011). The unique difficulties and challenges encountered by Generations 1 and 1.5 compared to their parents, Australian-born siblings as well as migrant youth from non-refugee backgrounds and non-migrant youth, are among the main reasons they were selected as the target population of the present research project.

Leading nationalities for humanitarian entrants: The study targeted refugee background youth from three migration regions of the world—Africa, the Middle East and South Asia, with around 200 participants from each migration group.

Initially, the study targeted refugee youth from countries within these three regions that represented the major source countries for humanitarian entrants to Australia, during the selected timeframe (Department of Immigration and Citizenship (DIAC), 2009; Department of Immigration and Multicultural Affairs (DIMA), 2007). The criteria for ethnicity was to be self-ascribed cultural / ethnic background. The migration regions and identified countries were:

- **Middle East** – Afghanistan, Iran, Iraq
- **South Asia** –Bhutan, Nepal, Myanmar/Burma, Pakistan
- **Africa** – Dem. Rep. of Congo, Rep. of Congo, Ethiopia, Somalia, Sudan, South Sudan

The 2011 and 2016 census data did not include visa status for people born overseas. Therefore, although not ideal, the best available method to ascertain the number of people from a refugee background in Australia was to consider whole population groups that were most likely to come to Australia as humanitarian entrants (Hugo et al., 2014).

According to the Department of Home Affairs (DOHA, 2018), the leading five countries of birth for persons granted visas under Australia's Humanitarian Program were Iraq, Syria, Afghanistan, Myanmar, and Bhutan. Other countries with significant numbers of refugees to Australia were, the Dem. Rep. of Congo, Eritrea, Ethiopia, South Sudan and Somalia. Whereas most of these countries were consistently among the leading 10 countries for humanitarian entrants to Australia since 2012-2013 (5 years), Iran was recognised in the top 10 list of refugee source countries until 2015-2016.

However, there was a possibility that the leading countries for refugee background youth aged 15-24 years and residing in South Australia could be different to the national trends depicted in the census of 2011 and 2016. Another consideration was the time lag of a few years between writing project proposal, project approval, and project commencement. Therefore, after further consultation among the Project Management Committee, the criterion for participants' cultural / ethnic background was revised to include Syria and Liberia (see ***Table 4*** below). Because migration trends are in constant flux and there is a real possibility of continuous changes to population trends, youth aged 15-24 years, born in countries outside those listed in ***Table 4*** were also deemed eligible to participate in the research project, as long as their self-identified cultural / ethnic background was within one of the three identified migration regions.

The three migration regions and countries identified for the project.

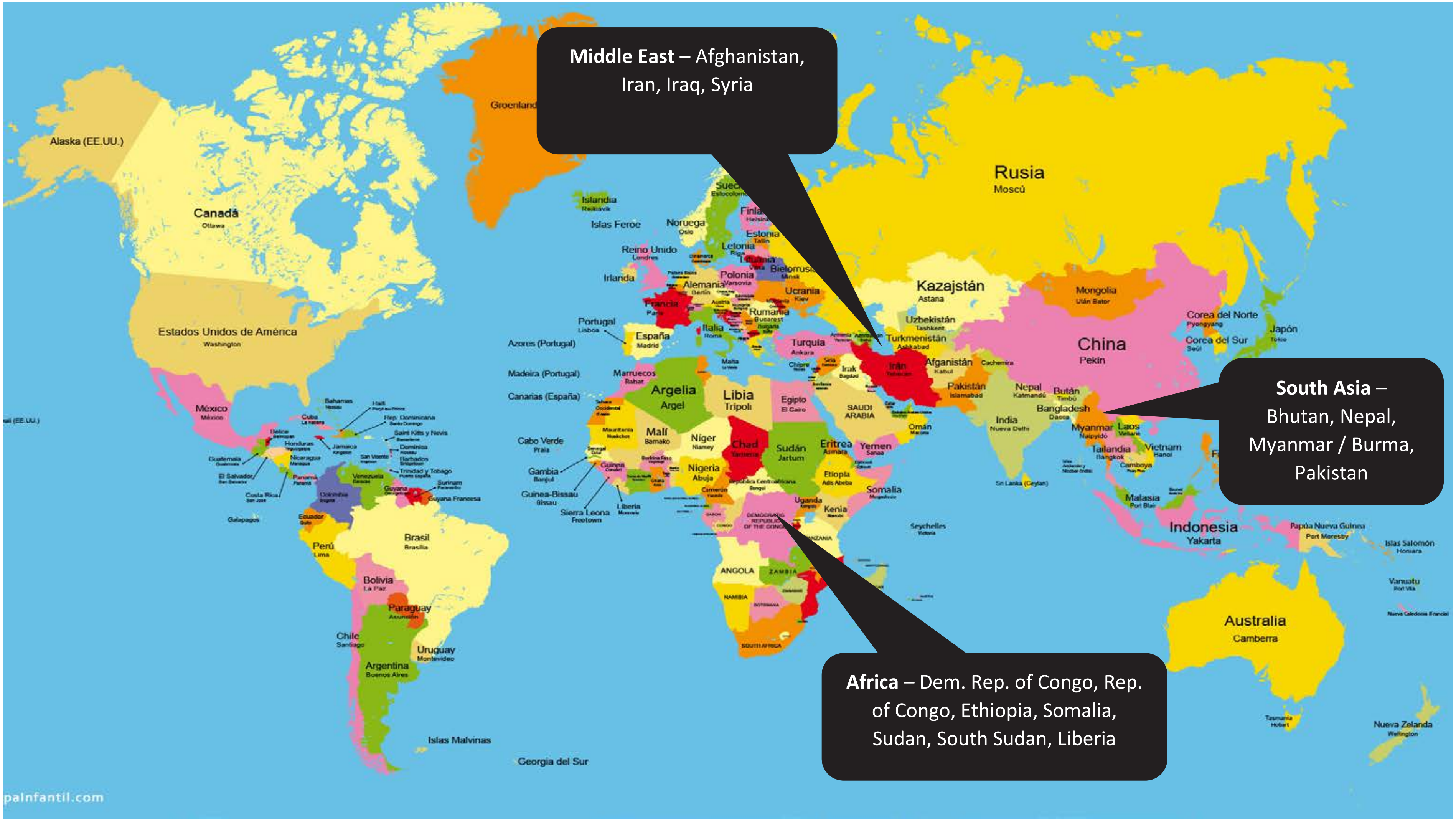

B. Refugee Youth 15-24 years – Australia and South Australia: Census Data

The first sub-section of this report section examines Census data over time for all youth aged 15 to 24 years, comparing figures for Australia and South Australia over three Census periods 2006, 2011 and 2016. Sub-sections Two and Three examine each nominated birthplace group individually with Section Two focusing on youth from each refugee birthplace country by region of SA over time and Section Three, on youth aged 15 to 24 years in South Australia, arriving between 2012 and 2016.

1. Refugee Youth by Region in Australia and South Australia

Table 1: Comparison of Youth Aged 15-24 years in 2016

Youth Aged 15-24 years in 2016	
Australia	2,704,269
South Australia	200,868
Australia - Refugee Country of Birth (COB)	64,914
South Australia - Refugee COB	5,485

Source: ABS Census Data 2016

Notes on Table 1:

- South Australia's youth aged 15 to 24 represent 7.43% of all youth in Australia.
- Youth from the ARC LP defined list of refugee countries of birth, aged 15 to 24 represent only 2.4% of all youth in Australia; whereas for South Australia youth from these defined refugee countries of birth represent a slightly higher proportion of the SA youth population at 2.7%.

Table 2: Comparison of Refugee Youth (15 to 24 Years) 2006, 2011 and 2016 for Australia and South Australia by Birthplace Region and Country

		2016		2011		2006	
**Regions**	**Refugee COB**	**Australia**	**South Australia**	**Australia**	**South Australia**	**Australia**	**South Australia**
**Africa**	**Sudan**	4288	283	4907	413	4648	465
	**South Sudan**	1513	155	866	91	*	*
	**Republic of Congo**	476	87	305	84	112	26
	**Dem. Republic of Congo**	1226	243	733	206	98	21
	**Ethiopia**	1940	176	1321	130	865	71
	**Somalia**	1220	59	1374	62	12126	48
	**Liberia**	561	103	748	148	565	121
	Africa Total	***11224***	***1106***	***10254***	***1134***	***7504***	***752***
**Middle East**	**Afghanistan**	11357	1830	7644	1091	4107	357
	**Iran**	5680	629	3776	411	3212	294
	**Iraq**	8881	243	8455	275	5986	158
	**Syria**	1511	126	513	26	542	27
	Middle East Total	***27429***	***2828***	***20388***	***1803***	***13847***	***836***
**South Asia**	**Myanmar**	4693	306	3026	126	1062	19
	**Bhutan**	432	53	499	165	21	0
	**Nepal**	11996	696	6469	226	1056	20
	**Pakistan**	9140	496	4740	215	2722	97
	South Asia Total	***26,261***	***1551***	***14,734***	***732***	***4861***	***136***
Total		**64914**	**5485**	**45376**	**3669**	**26212**	**1724**

** South Sudan was not a separate Country of Birth in the 2006 Census* *Source: ABS Census 2016, 2011 and 2006*

Figure 1: Refugee Youth (15 to 24 Years) 2006, 2011 and 2016 for Australia by Birthplace Country and Region

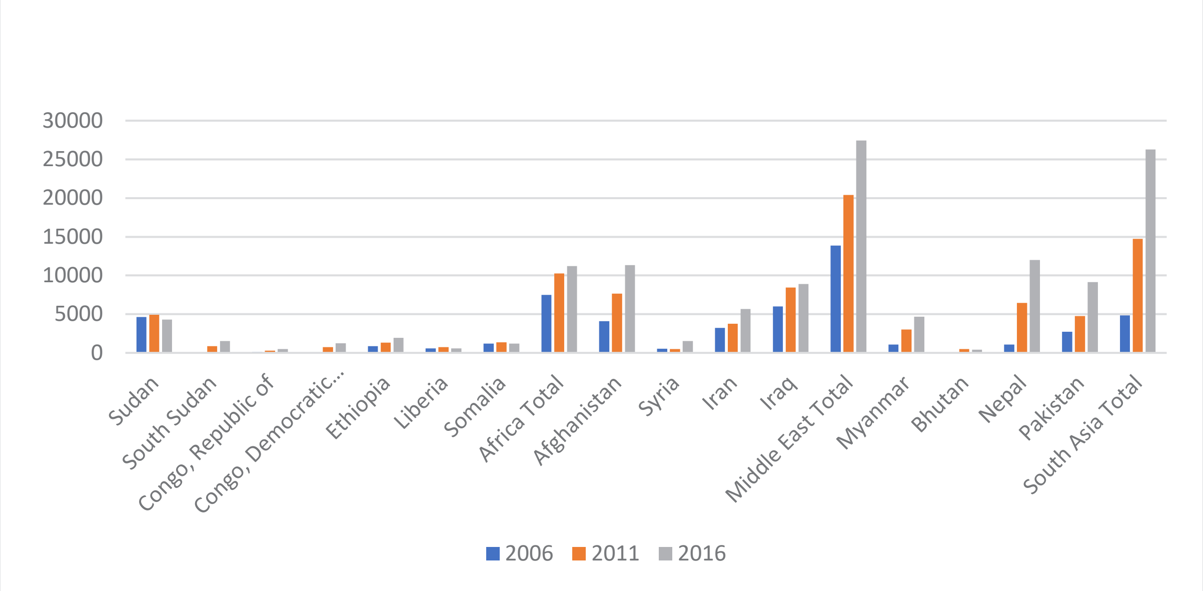

Source: ABS Census 2016, 2011 and 2006

Figure 2: Refugee Youth (15 to 24 Years) 2006, 2011 and 2016 for South Australia by Birthplace Country and Region

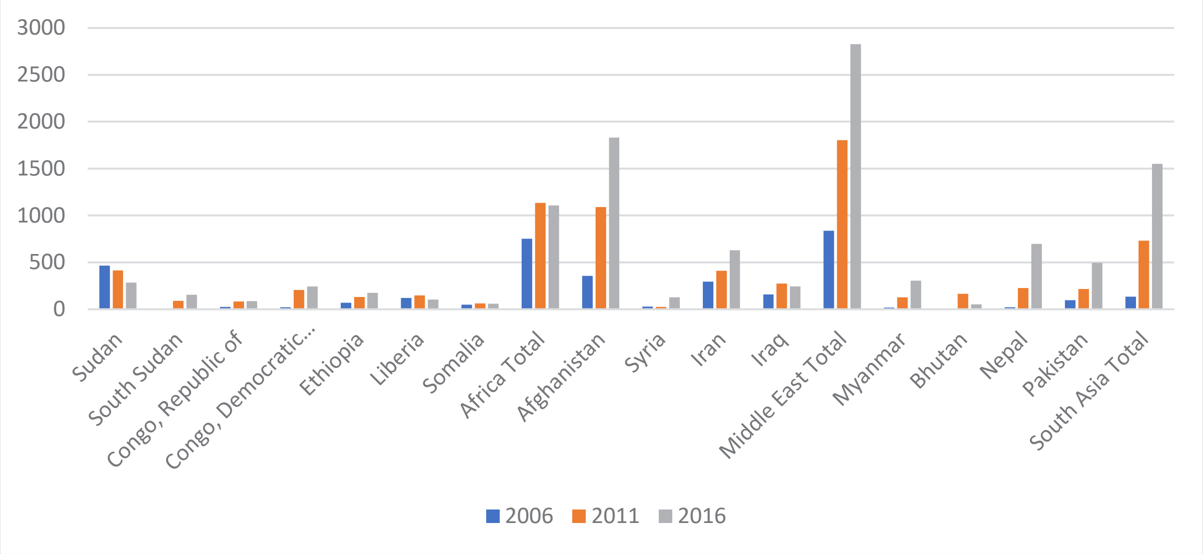

Source: ABS Census 2016, 2011 and 2006

Notes from Table 2, Figure 1 and Figure 2:

- South Australia shows a similar pattern of change to Australia over the past decade, with steadying numbers of refugee youth from the African countries, slight increases from the Middle East but the largest increases coming from the South East Asia region. In fact, the numbers for South Asia have risen from 493 youth in 2006 to almost 3,500 in 2016, an increase of 686%.

- However, while the trend is similar, there are distinct differences in the large increases from SE Asia in Australia and South Australia. The increase in Australia's refugee youth population from South Asia is a result of relatively equal increases across all birthplace groups; whereas in South Australia it can be attributed to significant increases in the numbers of youth coming from Nepal. In South Australia, for the Middle East region, the Afghanistan youth population has increased from 357 to over 1,800 over this decade, a more significant rise than for Australia as a whole.
- Figures 3 and 4 highlight the variations between national and state refugee youth population groups; emphasising the increasing significance of the South Asia birthplace groups at the national, and in particular, the state level.
- While the proportion of all youth from refugee backgrounds from the Middle East has declined slightly at a national level, we see a slight increase at the state level – this is mainly attributed to the increasing numbers of youth from Afghanistan.
- Youth arriving from the Sudan and Liberia have experienced decreases in population numbers over the decade, at both the national and state level, while numbers for Somalia have remained steady at the state level but decreased at the national level.

Figure 3: Percentage of Total Refugee Youth (15 to 24 years) by Region for Australia, 2006, 2011 and 2016

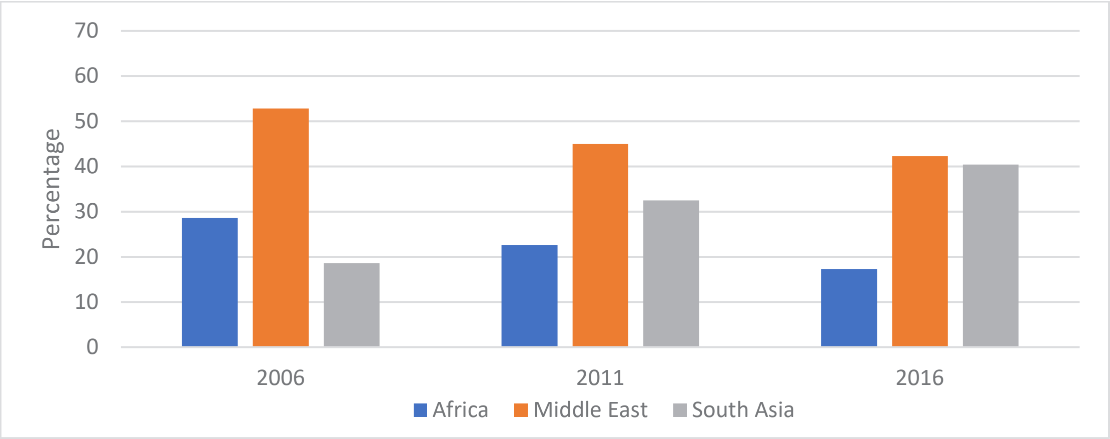

Source: ABS Census Data 2006, 2011 and 2016

Figure 4: Percentage of Total Refugee Youth (15 to 24 years) by Region for South Australia, 2006, 2011 and 2016

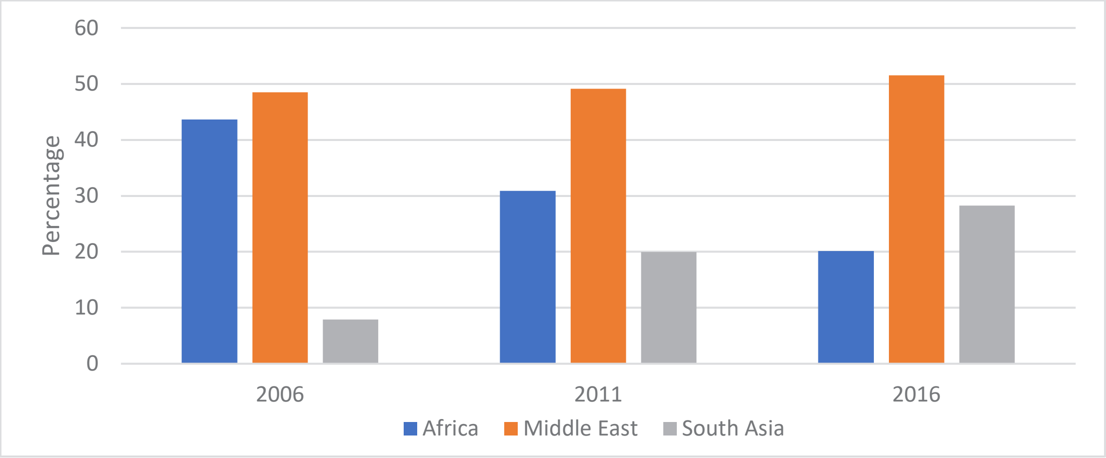

Source: ABS Census Data 2006, 2011 and 2016

2. South Australia: Analysis by Regions over Time

There are several things to note about Tables 3, 4 & 5, showing the distribution of refugee youth across the different regions of South Australia in 2006, 2011 and 2016:

- Between 2006 and 2011 the geography used by the ABS changed – thus you will note that the regional divisions of the state (highlighted in green on each table) differ between 2006 and 2011/2016. However, the area we call 'Adelaide', i.e.: the only major urban region in South Australia and the 'Rest of State' remain roughly the same and therefore there are not expected to be significant variations in the figures provided across years.
- Figures for the Adelaide region in 2011 and 2016 have been taken from the four regions used to define the greater Adelaide region from this time (highlighted in orange). The various regions are left as separate columns so that distribution across the Adelaide and Rest of State can be examined but please note that these figures have been summed in the columns "Adelaide' and 'Rest of State' – therefore total population figures for each birthplace group across the whole region must take this into account.
- The ABS randomises small cell numbers to ensure confidentiality of individual data – as such any cell in the table with numbers under 20 may not be accurate and should be used with caution.

Table 3: Youth 15-24 years from Refugee Countries of Birth by Regions in South Australia, 2006

	Adelaide	Rest of State'	Outer Adelaide	Yorke and Lower North	Murray Lands	South East	Eyre	Northern - SA	No Usual Address
**Sudan**	450	12	0	0	8	4	0	0	0
**South Sudan***									
**Congo**	26	0	0	0	0	0	0	0	0
**Dem Republic of Congo**	21	0	0	0	0	0	0	0	0
**Ethiopia**	71	3	0	0	3	0	0	0	0
**Liberia**	122	3	0	0	0	0	0	0	3
**Somalia**	49	0	0	0	0	0	0	0	0
Africa Total	***739***	***18***	***0***	***0***	***11***	***4***	***0***	***0***	***3***
**Afghanistan**	334	16	0	0	16	0	0	0	0
**Syria**	27	0	0	0	0	0	0	0	0
**Iran**	291	3	3	0	0	0	0	0	0
**Iraq**	155	3	0	0	3	0	0	0	0
Middle East Total	***807***	***22***	***3***	***0***	***19***	***0***	***0***	***0***	***0***
**Burma (Myanmar)**	14	0	0	0	0	0	0	0	0
**Bhutan**	0	0	0	0	0	0	0	0	0
**Nepal**	16	0	0	0	0	0	0	0	0
**Pakistan**	93	10	3	0	0	0	0	5	2
South Asia Total	***123***	***10***	***3***	***0***	***0***	***0***	***0***	***5***	***2***
**Total**	**1669**	**50**	**6**	**0**	**30**	**4**	**0**	**5**	**5**

Note: There is no recognised country 'South Sudan' in 2006 *Source: ABS 2006 Census of Population and Housing*

Table 4: Youth 15-24 years from Refugee Countries of Birth by Regions in South Australia, 2011

	Adelaide - Central and Hills	Adelaide - North	Adelaide - South	Adelaide - West	Adelaide	Rest of State	Barossa - Yorke - Mid North	South Australia - Outback	South Australia - South East	No Usual Address (SA)
**Sudan**	56	202	33	107	398	9	0	0	9	0
**South Sudan**	22	42	3	18	85	6	0	0	6	0
**Republic of Congo**	10	58	7	8	83	7	0	0	7	0
**Dem Rep. of Congo**	16	91	30	48	185	18	0	0	18	0
**Ethiopia**	26	39	24	34	123	6	3	0	3	0
**Liberia**	15	62	10	59	146	3	0	0	0	3
**Somalia**	3	3	4	49	59	4	0	0	0	4
Africa Total	***148***	***497***	***111***	***323***	***1079***	***53***	***3***	***0***	***43***	***7***
**Afghanistan**	66	706	38	190	1000	87	0	0	87	0
**Syria**	0	8	3	11	22	0	0	0	0	0
**Iran**	97	171	83	57	408	3	0	0	3	0
**Iraq**	19	156	33	62	270	5	0	0	5	0
Middle East Total	***182***	***1041***	***157***	***320***	***1,700***	***95***	***0***	***0***	***95***	***0***
**Burma (Myanmar)**	7	56	3	33	99	16	0	0	16	0
**Bhutan**	12	143	3	8	166	0	0	0	0	0
**Nepal**	62	81	40	45	228	0	0	0	0	0
**Pakistan**	29	53	90	39	211	7	0	4	0	3
South Asia Total	***110***	***333***	***136***	***125***	***704***	***23***	***0***	***4***	***16***	***3***
**Total**	440	1871	404	768	3483	171	3	4	154	10

Source: ABS 2011 Census of Population and Housing

Table 5: Youth 15-24 years from Refugee Countries of Birth by Regions in South Australia, 2016

	Adelaide - Central and Hills	Adelaide - North	Adelaide - South	Adelaide - West	Adelaide	Rest of State	Barossa - Yorke - Mid North	South Australia - Outback	South Australia - South East	No Usual Address (SA)
**Sudan**	26	125	10	112	273	5	0	0	4	0
**South Sudan**	18	125	5	13	161	0	0	0	0	0
**Republic of Congo**	8	55	5	12	80	11	0	0	7	0
**Dem Rep. of Congo**	20	134	35	33	222	32	0	5	22	0
**Ethiopia**	29	31	31	70	161	24	5	0	10	4
**Liberia**	9	55	0	37	101	0	0	0	0	0
**Somalia**	0	7	12	45	64	0	0	0	0	0
Africa Total	***110***	***532***	***98***	***322***	***1062***	***72***	***5***	***5***	***43***	***4***
**Afghanistan**	97	1444	45	165	1751	91	0	3	83	0
**Syria**	9	65	21	23	118	4	0	0	4	0
**Iran**	121	325	89	86	621	3	0	0	3	0
**Iraq**	21	145	13	58	237	0	0	0	0	0
Middle East Total	***248***	***1979***	***168***	***332***	***2,727***	***105***	***0***	***3***	***90***	***0***
**Myanmar**	17	217	0	36	270	39	0	0	32	0
**Bhutan**	0	50	6	0	56	0	0	0	0	0
**Nepal**	50	422	107	118	697	0	0	0	0	0
**Pakistan**	81	183	120	98	482	23	0	4	12	0
South Asia Total	***148***	***872***	***233***	***252***	***1505***	***62***	***0***	***4***	***44***	***0***
**Total**	506	3383	499	906	5294	232	5	12	177	4

Source: ABS 2016 Census of Population and Housing

Notes of Regional Tables:

- Note the significant increase in the number of refugee youth living outside of the metropolitan area between 2006 and 2016 (from 50 to 232).
- This regional youth population is mainly centred on the Limestone Coast/South East region. This is to be expected as this is the only planned settlement site for refugee families in regional SA.
- In 2011 and 2016 the northern and western regions of Adelaide (particularly the northern areas) remain the most highly populated by refugee youth, and most increases have been seen in these regions over this period, while the southern and eastern regions of Adelaide have remained steady.

3. Profile of Youth 15-24 years by Refugee Birthplace Groups for South Australia (Arrived 2012–2016)

The following sections explore each of these nominated refugee birthplace groups in more detail using the 2016 ABS Census of Population and Housing data. Data is presented on English proficiency, language spoken at home, citizenship, and employment and student status for each birthplace group. It must be noted that when examining small cohorts such as this, totals may vary between tables due to the ABS randomising small cell numbers to ensure confidentiality.

3.1. Nominated Refugee Countries in the Africa Region

Seven countries make up the refugee birthplace groups for the Africa Region – Sudan, South Sudan, the Republic of Congo, the Democratic Republic of Congo, Ethiopia, Liberia and Somalia. Together these countries represented just over 20% of South Australia's youth population from nominated refugee countries; a decrease from 43.6% in 2006. However, in terms of raw numbers the youth population from the African region has increased at both the Australian level (from 7,504 in 2006 to 11,224 in 2016) and the South Australian level (from 752 in 2006 to 1,106 in 2016).

3.1.1 Sudan

The total Sudanese born population in South Australia decreased from 1,416 people in 2011 to 992 people in 2016. This equates with an average annual population change of -6.9%. Table 6 shows that youth aged 15 to 24 years represented 28.5% of the total Sudanese born population in South Australia in 2016, just slightly less than the 25-34 year old cohort. While this age cohort represented over 28% of all arrivals during the 2012-2016 period, overall only 10% of this youth cohort had arrived in South Australia in the 2012-2016 time period.

Table 6: Overview of the Sudanese Population, South Australia

Age	Arrived 2012-2016	Total Pop 2016	% Arrived 2012-16
**0-14**	22	87	25.3
**15-24**	29	283	10.2
**25-34**	24	290	8.3
**35-44**	21	173	12.1
**45+**	6	159	3.8
**Total**	102	992	10.3

Source: ABS, 2016 Census, Usual Place of Address

One hundred percent of the Sudanese-born youth cohort who arrived during this period were living in the Greater Adelaide region. Of those in the 15-24-year-old cohort who arrived in the 2012-2016 period, just over half (57.7%) speak English well or very well. The top languages spoken at home were Arabic and Dinka. The top two nominated religions were Western Catholic and Islam. 100% of the 26 who responded were not Australian citizens at the time of the 2016 Census. Of the 29 Sudanese-born youth who arrived between 2012 and 2016, 27[2] were not in the labour force at the time of the 2016 Census, as shown in Table 7.

[2] The ABS randomises small cell numbers and therefore caution must be exercised when evaluating this data.

Table 7: Employment Status, Newly Arrived (2012-2016) Sudanese Youth, South Australia

Employment Status	Number	Percent
Not in The Labour Force	27	100.0
In the Labour Force	0	0.0

Source: ABS, 2016 Census, Usual Place of Address

Table 8: Education Participation, Newly Arrived (2012-2016) Sudanese Youth, South Australia

FT/PT Student Status	Number	Percent
Not attending	4	19
Full-time student	17	81
Part-time student	0	0
Total (not incl Not Stated)	21	100

Source: ABS, 2016 Census, Usual Place of Address

Table 8 highlights that most of this cohort were attending school, with over 80% full time students. As Table 9 shows, 60% of this group were attending secondary school, with none attending University. This current schooling status needs to be taken into account when looking at Table 10, where only 35% of this cohort have Year 11 or 12 equivalent. As 60% are still in secondary school it can be assumed that this proportion will increase over time.

Table 9: Type of Educational Institution Attending, Newly Arrived (2012-2016) Sudanese Youth, South Australia

	Number	Percent
Secondary	15	60
Technical or Further Education	6	24
University or Other Tertiary	0	0
Other	0	0
Not Stated	0	0
Not Applicable	4	16
Total	25	100

Source: ABS, 2016 Census, Usual Place of Address

Table 10: Highest Level of Schooling Completed in 2016, Newly Arrived (2012-2016) Sudanese Youth, South Australia

	Number		Percent
Year 12 or equivalent	3		15
Year 11 or equivalent	4		20
Year 10 or equivalent	3		15
Year 9 or equivalent	0		0
Year 8 or below	10		50
Did not go to school	0		0
Not stated	0		0
Total	20		100

Source: ABS, 2016 Census, Usual Place of Address

3.1.2 South Sudan

The average annual growth rate from 2011-2016 of those born in South Sudan living in South Australia was 17.9%. The total population rose from 389 persons in 2011 to 887 in 2016. Youth aged 15 – 24 years represented 21.9% of the total South Sudanese 2016 population; with almost 22% of this age cohort having arrived between 2012 and 2016. This age cohort represented almost half of all arrivals from South Sudan during this period.

Table 11: Overview of the South Sudanese Population, South Australia

Age	Arrived 2012-2016	Total Pop 2016	% Arrived 2012-16
**0-14**	8	46	17.4
**15-24**	34	155	21.9
**25-34**	11	284	3.9
**35-44**	3	269	1.1
**45+**	6	133	4.5
**Total**	62	887	7.0

Source: ABS, 2016 Census, Usual Place of Address

As with the Sudanese population, 100% of this 15-24 age cohort who arrived between 2012 and 2016 were living in the Greater Adelaide region, with 28 of these living in the northern region of Adelaide.

Within this youth cohort, arriving between 2012 and 2016, 65.2% spoke English well or very well at the time of the 2016 Census. The main languages spoken at home were Dinka and Acholi and the main religions stated were Anglican and Western Catholic. One hundred percent of the 29 who responded were not Australian citizens at the time of the 2016 Census.

Table 12 shows that 100% of this youth cohort arriving between 2012 and 2016 were not in the labour force at the time of the 2016 Census. Almost two thirds of this group (65%) were, however, attending school and, as shown in Table 13, most of those were full time students.

Table 12: Employment Status, Newly Arrived (2012-2016) South Sudanese Youth, South Australia

Employment Status	Number	Percent
Not in The Labour Force	20	100.0
In the Labour Force	0	0.0

Source: ABS, 2016 Census, Usual Place of Address

Table 13: Education Participation, Newly Arrived (2012-2016) South Sudanese Youth, South Australia

FT/PT Student Status	Number	Percent
**Not attending**	12	35.3
**Full-time student**	18	52.9
**Part-time student**	4	11.8
**Total (not incl NS)**	34	100.0

Source: ABS, 2016 Census, Usual Place of Address

Table 14 shows that most of this cohort were in Secondary School, with only a small number attending other forms of further education (such as TAFE). No one from this cohort was attending University.

Table 14: Type of Educational Institution Attending, Newly Arrived (2012-2016) South Sudanese Youth, South Australia

	Number	Percent
**Secondary**	13	44.8
**Technical or Further Education**	4	13.8
**University or other Tertiary**	0	0.0
**Other**	0	0.0
**Not Stated**	0	0.0
**Not Applicable**	12	41.4
**Total**	29	100.0

Source: ABS, 2016 Census, Usual Place of Address

As shown previously, while only nine people in this youth cohort, arriving between 2012 and 2016, had completed year 11 or 12 at secondary school it must be remembered that 13 were still attending Secondary School at the time of the 2016 Census.

Table 15: Highest Level of Schooling Completed in 2016, Newly Arrived (2012-2016) South Sudanese Youth, South Australia

	Number	Percent
**Year 12 or equivalent**	5	35.7
**Year 11 or equivalent**	4	28.6
**Year 10 or equivalent**	0	0.0
**Year 9 or equivalent**	0	0.0
**Year 8 or below**	5	35.7
**Did not go to school**	0	0.0
**Not stated**	0	0.0
**Total**	14	100.0

Source: ABS, 2016 Census, Usual Place of Address

3.1.3 Republic of Congo

The total population of the Republic of Congo experienced a small increase between 2011 (207 people) and 2016 (238 people). This represented an average annual growth rate in the total population of just 2.8% between 2011 and 2016. Most of this increase, as shown in Table 16, was in the 15 to 24 years and 25-34 years age groups, with 29 new arrivals between 2012 and 2016. Those aged 15 to 24 years represented almost half of all arrivals during this time and made up 38% of the total Republic of Congo population at the time of the 2016 Census.

Table 16: Overview of the Republic of Congo Population, South Australia

Age	Arrived 2012-2016	Total Pop 2016	% Arrived 2012-16
**0-14**	7	41	17.1
**15-24**	15	91	16.5
**25-34**	14	58	24.1
**35-44**	0	24	0.0
**45+**	0	24	0.0
**Total**	36	238	15.1

Source: ABS, 2016 Census, Usual Place of Address

One hundred percent of this youth cohort arriving between 2012 and 2016 were living in the Greater Adelaide region at the time of the 2016 Census and none had become Australian Citizens. One hundred percent of this cohort also spoke English well or very well and the main language spoken at home was Swahili. Christian (not defined) was the main nominated religion.

Population numbers were too small (n=15) within this cohort for any meaningful data to be drawn from the ABS on employment and education.

3.1.4 Democratic Republic of Congo

The total population of the Democratic Republic of Congo was 674 people in 2016, an increase from 544 people in 2011. This represents an average annual growth rate of 4.38 percent. Of this total population in 2016, youth aged 15 to 64 years represented 36% of the total population; with 24.5% of the 15–24 year old population (60 persons) having arrived between 2012 and 2016 – see Table 17 . This was just over a third of all arrivals during this time period.

Table 17: Overview of the Democratic Republic of Congo Population, South Australia

Age	Arrived 2012-2016	Total Pop 2016	% Arrived 2012-16
**0-14**	37	77	48.1
**15-24**	60	244	24.6
**25-34**	36	163	22.1
**35-44**	21	97	21.6
**45+**	17	93	18.3
**Total**	171	674	25.4

Source: ABS, 2016 Census, Usual Place of Address

Table 18 shows that of this 15-24 years of age cohort who arrived between 2012 and 2016, 0ver 85 percent lived in the Greater Adelaide region, with the majority of those living in the north. A much smaller proportion were living in rural and regional South Australia.

Table 18: Place of Residence for Newly Arrived (2012-2016) Congolese (DRC) Youth, South Australia

Region	Number	Percent
**Greater Adelaide**	53	85.5
**Rest of State**	9	14.5
**Total**	62	100.0
**Adelaide –Central & Hills**	7	
**Adelaide -North**	43	
**Adelaide -South**	12	
**Adelaide -West**	5	

Source: ABS, 2016 Census, Usual Place of Address

Almost three quarters (72.3%) of this population cohort spoke English well or very well, with Swahili and French the main languages spoken at home. No-one in this cohort of new arrivals was an Australian Citizen at the time of the 2016 Census and Christianity (not defined) was the main nominated religion.

One hundred percent of this cohort were not in the labour force at the time of the 2016 Census, and as seen in Table 19, just over half (55%) were in some form of education. Table 20 shows that of this population participating in education over half were in secondary school and almost a quarter were at some form of technical college or further education. None were attending University.

Table 19: Education Participation, Newly Arrived (2012-2016) Congolese (DRC) Youth, South Australia

FT/PT Student Status	Number	Percent
**Not attending**	31	44.9
**Full-time student**	29	42.0
**Part-time student**	9	13.0
**Total (not incl Not Stated)**	69	100.0

Source: ABS, 2016 Census, Usual Place of Address

Table 20: Type of Educational Institution Attending, Newly Arrived (2012-2016) Congolese (DRC) Youth, South Australia

	Number	Percent
**Secondary**	27	55.1
**Technical or Further Education**	12	24.5
**University or other Tertiary**	0	0.0
**Other**	4	8.2
**Not Stated**	3	6.1
**Not Applicable**	3	6.1
**Total**	49	100.0

Source: ABS, 2016 Census, Usual Place of Address

Table 21: Highest Level of Schooling Completed in 2016, Newly Arrived (2012-2016) Congolese (DRC) Youth, South Australia

	Number	Percent
**Year 12 or equivalent**	15	34.1
**Year 11 or equivalent**	10	22.7
**Year 10 or equivalent**	10	22.7
**Year 9 or equivalent**	5	11.4
**Year 8 or below**	4	9.1
**Did not go to school**	0	0.0
**Not stated**	0	0.0
**Total**	44	100.0

Source: ABS, 2016 Census, Usual Place of Address

3.1.6 Ethiopia

The total population of persons born in Ethiopia in South Australia had an average annual growth rate of 6.36% between 2011 and 2016. The total population increased from 582 persons to 792 persons in this time period. Youth aged 15 to 24 years represented over 20% of the total population in 2016; with over 35% of this cohort having arrived between 2012 and 2016, as shown in Table 22.

Table 22: Overview of the Ethiopian Population, South Australia 2016

Age	Arrived 2012-2016	Total Pop 2016	% Arrived 2012-16
**0-14**	63	113	55.8
**15-24**	60	170	35.3
**25-34**	48	153	31.4
**35-44**	37	170	21.8
**45+**	16	186	8.6
**Total**	224	792	28.3

Source: ABS, 2016 Census, Usual Place of Address

One hundred percent of this 15-24 years, new arrival population was living in the Greater Adelaide region at the time of the 2016 Census. As with other refugee youth from the African region none of the recent arrivals were Australian citizens. Over three quarters (75.5%) of this youth cohort from Ethiopia who arrived between 2012 and 2016 spoke English well or very well and the three main languages spoken at home were Amharic, English and Oromo. Ethiopian Islam and Christian (not defined) were the two most nominated religions within this population cohort.

Table 23 and Table 24 show that about 18% of this newly arrived youth population were in the labour force while 75% nominated that they were either a full time or part time student.

Table 23: Employment Status, Newly Arrived (2012-2016) Ethiopian Youth, South Australia

Employment Status	Number	Percent
**Not in The Labour Force**	32	74.4
**In the Labour Force**	11	25.6

Source: ABS, 2016 Census, Usual Place of Address

Table 24: Education Participation, Newly Arrived (2012-2016) Ethiopian Youth, South Australia

FT/PT Student Status	Number	Percent
**Not attending**	9	16.7
**Full-time student**	39	72.2
**Part-time student**	6	11.1
**Total (not incl NS)**	54	100.0

Source: ABS, 2016 Census, Usual Place of Address

Most of those who nominated being a student (57.7%) were in Secondary School, with over 15% attending University and almost a further 10% attending another form of further education, such as TAFE or vocational college – see Table 25.

Table 25: Type of Educational Institution Attending, Newly Arrived (2012-2016) Ethiopian Youth, South Australia

	Number	Percent
**Secondary**	30	57.7
**Technical or Further Education**	5	9.6
**University or other Tertiary**	8	15.4
**Other**	0	0.0
**Not Stated**	0	0.0
**Not Applicable**	9	17.3
**Total**	52	100.0

Source: ABS, 2016 Census, Usual Place of Address

As with the other population groups, the smaller numbers having completed the higher years of schooling, as shown in Table 26, needs to be contextualised with the number stating that they were still in Secondary School at the time of the 21016 Census.

Table 26: Highest Year of Schooling Completed in 2016, Newly Arrived (2012-2016) Ethiopian Youth, South Australia

	Number	Percent
**Year 12 or equivalent**	12	24
**Year 11 or equivalent**	15	30
**Year 10 or equivalent**	5	10
**Year 9 or equivalent**	6	12
**Year 8 or below**	3	6
**Did not go to school**	9	18
**Not stated**	0	0
**Total**	50	100

Source: ABS, 2016 Census, Usual Place of Address

3.1.7 Liberia

The total population of Liberia had a very small increase between 2011 (541 persons) and 2016 (567 persons); this represents an annual average growth rate of only 0.94%. Youth aged 15 to 24 years represented approximately 18% of the total population in 2016, and just over 18% of the total new arrivals for this birthplace group between 2012 and 2016. New arrivals were 11.8% of the total population aged 15-24 years at the 2016 Census.

Table 27: Overview of the Liberian Population, South Australia 2016

Age	Arrived 2012-2016	Total Pop 2016	% Arrived 2012-16
**0-14**	9	18	50.0
**15-24**	12	102	11.8
**25-34**	20	195	10.3
**35-44**	24	152	15.8
**45+**	0	93	0.0
**Total**	65	567	11.5

Source: ABS, 2016 Census, Usual Place of Address

One hundred percent of this newly arrived youth population were living in the Greater Adelaide region in 2016 and no one was an Australian citizen. Of this population group, almost all (95.8%) spoke English well or very well, with the main languages spoken at home English and Liberian English. The main nominated religion for this group was Baptist. Almost 40% of this cohort nominated being in the labour force at the time of the 2016 Census, and over 93% were in school (86.7% in fulltime education).

As with the Republic of Congo cohort, the population numbers for youth aged 15 to 24 years who arrived between 2012 and 2016 from Liberia were too small (n=12) for any meaningful data to be drawn from the ABS on employment and education.

3.1.7 Somalia

The total population of people born in Somalia in South Australia increased slightly from 252 in 2011 to 264 in 2016. This represents an average annual growth rate of 0.93%. Of this total population youth aged 15 to 24 years represented 21%, with 22 of the 56 persons (39.3%) having arrived between 2012 and 2106. This represents 31.8% of all arrivals from Somalia during this period.

Table 28: Overview of the Somalian Population, South Australia 2016

Age	Arrived 2012-2016	Total Pop 2016	% Arrived 2012-16
**0-14**	21	27	77.8
**15-24**	22	56	39.3
**25-34**	18	67	26.9
**35-44**	8	50	16.0
**45+**	0	64	0.0
**Total**	69	264	26.1

Source: ABS, 2016 Census, Usual Place of Address

For this newly arrived 15-24 years population 100% were living in the Greater Adelaide region, with most living in Adelaide-West. No youth who arrived in the 2012 to 2016 period had taken out Australian Citizenship and 77% were able to speak English well or very well. The main language spoken at home was Somali and the main religion was Islam.

Almost 90% of this cohort were not in the labour force at the time of the 2016 Census and 69% were full-time students (no one reported being a part time student).

Numbers for different types of educational setting and levels of education were too low to be considered reliable.

3.1.8 Overview of Refugee Youth Arriving between 2012 and 2016 in the African Region

The preceding tables highlight the predominance of youth in the migration figures from refugee countries across Africa. While youth aged 15 to 24 years in the seven African refugee nominated countries comprised 24.9% of the total population, this age cohort made up 31.8% of all arrivals between 2012 and 2016 (see Table 29 below).

The largest numbers of youth in this cohort came from the Democratic Republic of Congo and Ethiopia during the time period, but these also represented the largest numbers of new arrivals from these countries overall.

Table 29: Overview of Population for Refugee Nominated Countries across the Africa Region for South Australia, 2016

	15-24yrs, Arrived 2012-2016	Total Pop 15-24yrs 2016	Total Pop 2016	Total Pop Arrived 2012-2016
**Sudan**	29	283	992	102
**South Sudan**	34	155	887	62
**Republic of Congo**	15	244	238	36
**Democratic Republic of Congo**	60	91	674	171
**Ethiopia**	60	170	792	224
**Liberia**	12	102	567	65
**Somalia**	22	56	264	69
**Africa Region**	**232**	**1101**	**4414**	**729**

Source: ABS, 2016 Census, Usual Place of Address

There were no newly arrived youth from the African region who had taken up Australian citizenship at the time of the 2016 Census. Considering this data is only looking at those who arrived between 2012 and 2016 this is perhaps not entirely surprising, although other birthplace regions do show small numbers of citizenship for this cohort.

Only three countries were reported by the Census to have any youth aged 15 to 24 years who arrived between 2012 and 2016 in the labour force: Ethiopia (25%), Liberia (10%) and Somalia (11%). However, it must be remembered that the numbers for Liberia and Somalia are very small and therefore the data is unreliable. What is interesting to note is that for two of these countries (Liberia and Ethiopia) English is nominated as a language spoken at home. This suggests that prior proficiency in English may be helping this cohort to find employment earlier than other population groups. Further examination of this data would provide more information on whether this work is full time or part time and the nature of the industry of employment.

Levels of proficiency in English language were generally high across all birthplace groups for this 15 to 24 y/o population – ranging from 57.7% and 65% in Sudan and South Sudan respectively to 95.8% and 100% in Liberia and the Republic of Congo respectively. Of course, these figures are based on self-reported assessments of English language proficiency and not on any standardised test scores; thus actual levels of proficiency may vary more than this.

3.2 Nominated Refugee Countries in the Middle East Region

Four countries make up the refugee birthplace groups for the Middle East Region – Afghanistan, Syria, Iran and Iraq. In 2006 youth aged 15 to 24 years from nominated refugee countries in this region made up 52.8% of all refugee youth in Australia and 48.5% in South Australia. By 2016, this group were 42.3% of the national refugee youth population but had increased to form 51.6% of South Australia's refugee youth population aged 15 to 24 years. This was mainly attributed to the increasing numbers of youth from Afghanistan settling in South Australia. In terms of actual population numbers, there have been increases at both the national and state levels. For Australia the population numbers for youth aged 15 to 24 years has almost doubled, from 13,847 in 2006 to 27,429 in 2016. For South Australia this increase saw the youth population for this region more than triple, from 836 in 2006 to 2,828 in 2016.

3.2.1 Afghanistan

The total population for the Afghanistan birthplace group in South Australia increased from 3,288 in 2011 to 6,284 in 2016. This represented an average annual growth rate of 13.83%. Table 30 shows that youth aged 15 to 24 years represented 28.9% of the total population in 2016 and 28.4% of all new arrivals between 2012 and 2016. In 2016, these new arrivals age 15 to 24 years comprised 41.5% of all youth from Afghanistan.

Table 30: Overview of the Afghani Population, South Australia 2016

Age	Arrived 2012-2016	Total Pop 2016	% Arrived 2012-16
**0-14**	433	565	76.6
**15-24**	755	1819	41.5
**25-34**	593	1510	39.3
**35-44**	459	1244	36.9
**45+**	419	1146	36.6
**Total**	2659	6284	42.3

Source: ABS, 2016 Census, Usual Place of Address

Table 31 highlights the settlement patterns of this newly arrived cohort of youth from Afghanistan, with 94.6% residing in the Greater Adelaide region and only 40 youth residing in rural or regional South Australia. Of those in the Greater Adelaide region they predominately have settled in the Adelaide-North area.

Table 31: Place of Residence for Newly Arrived (2012-2016) Afghani Youth, South Australia

Region	Number	Percent
**Greater Adelaide**	707	94.6
**Rest of State**	40	5.4
**Total**	747	100.0
Adelaide –Central & Hills	39	
Adelaide -North	590	
Adelaide -South	28	
Adelaide -West	39	

Source: ABS, 2016 Census, Usual Place of Address

Just over 70% of this youth cohort, who arrived between 2012 and 2016, spoke English well or very well; with the main languages spoken at home Dari and Hazaraghi. The main religion was Islam. A small proportion of this newly arrived Afghani youth population had taken out Australian citizenship (see Table 32 below).

Table 32: Citizenship, Newly Arrived (2012-2016) Afghani Youth, South Australia

Citizenship	Number	Percent
Australian	18	2.4
Not Australian	709	96.2
Not stated	10	1.4
Total	737	100.0

Source: ABS, 2016 Census, Usual Place of Address

While the majority of this newly arrived Afghani youth population were not in the labour force (83%), as shown in Table 33, 123 persons from this cohort were recorded as being in the workforce at the time of the 2016 Census. However, to counter the small percentage who are in the workforce Table 34 shows that over 63% were in some form of education – the majority of them are full time students. In Table 35 we see that most of these students are at Secondary School or some form of vocational or technical training while less than five percent are at University.

Table 33: Employment Status, Newly Arrived (2012-2016) Afghani Youth, South Australia

Employment Status	Number	Percent
Not in The Labour Force	602	83.0
In the Labour Force	123	17.0

Source: ABS, 2016 Census, Usual Place of Address

Table 34: Education Participation, Newly Arrived (2012-2016) Afghani Youth, South Australia

FT/PT Student Status	Number	Percent
Not attending	263	35.6
Full-time student	435	58.9
Part-time student	40	5.4
Total (not incl NS)	738	100.0

Source: ABS, 2016 Census, Usual Place of Address

Table 35: Type of Educational Institution Attending, Newly Arrived (2012-2016) Afghani Youth, South Australia

	Number	Percent
Secondary	270	36.9
Technical or Further Education	84	11.5
University or other Tertiary	32	4.4
Other	28	3.8
Not Stated	55	7.5
Not Applicable	263	35.9
Total	732	100.0

Source: ABS, 2016 Census, Usual Place of Address

The interesting point to note in Table 36 below is the high proportion of this newly arrived youth cohort who stated that they 'did not go to school' (126 persons or 16.6% of this cohort). This is by the far the highest number and proportion of any of the refugee birthplace groups in this study. To balance this, 167 (22.1%) had completed Year 12 by the time of the 2016 Census.

Table 36: Highest Year of Schooling Completed as of 2016, Newly Arrived (2012-2016) Afghani Youth, South Australia

	Number	Percent
**Year 12 or equivalent**	167	22.1
**Year 11 or equivalent**	135	17.8
**Year 10 or equivalent**	139	18.4
**Year 9 or equivalent**	69	9.1
**Year 8 or below**	110	14.5
**Did not go to school**	126	16.6
**Not stated**	11	1.5
**Total**	757	100.0

Source: ABS, 2016 Census, Usual Place of Address

3.2.2 Syria

The Syrian population in South Australia increased dramatically between 2011 (234 persons) to 2016 (812 persons), an obvious by-product of the recent conflicts in Syria. This represented an average annual growth rate increase of 28.3% over this time period. Youth aged 15 to 24 years only represented 17% of this newly arrived cohort; however, youth who arrived between 2012 and 2016 represented over 70% of all youth in this age cohort in 2016, as shown in Table 37.

Table 37: Overview of the Syrian Population, South Australia 2016

Age	Arrived 2012-2016	Total Pop 2016	% Arrived 2012-16
**0-14**	230	264	87.1
**15-24**	89	127	70.1
**25-34**	72	108	66.7
**35-44**	93	147	63.3
**45+**	33	166	19.9
**Total**	517	812	63.7

Source: ABS, 2016 Census, Usual Place of Address

As with other birthplace groups, the majority of this youth population was residing in the Greater Adelaide region (almost 97%), with most of that population in the Adelaide-North area. Six newly arrived youth from this cohort had Australian Citizenship. Less than 24% of this newly arrived youth cohort could speak English well or very well at the time of the 2016 Census. The main language spoken at home was Arabic and the main religion for this cohort was Islam.

Over 96% of newly arrived youth were not in the labour force at the time of the 2016 Census although this is countered by the very high proportion of this group who nominated still having student status, as shown below in Table 38. Those who are students are mainly at secondary and vocational education, with no one from this cohort nominating attending University (Table 39).

Table 38: Education Participation, Newly Arrived (2012-2016) Syrian Youth, South Australia

FT/PT Student Status	Number	Percent
**Not attending**	0	0.0
**Full-time student**	60	75.9
**Part-time student**	19	24.1
**Total (not incl NS)**	79	100.0

Source: ABS, 2016 Census, Usual Place of Address

Table 39: Type of Educational Institution Attending, Newly Arrived (2012-2016) Syrian Youth, South Australia

	Number	Percent
**Secondary**	47	57.3
**Technical or Further Education**	12	14.6
**University or other Tertiary**	0	0.0
**Other**	0	0.0
**Not Stated**	4	4.9
**Not Applicable**	19	23.2
**Total**	82	100.0

Source: ABS, 2016 Census, Usual Place of Address

Table 40 shows that over a third of this newly arrived youth cohort had completed Year 12 or its equivalent by the 2016 Census. The remaining levels of education may represent those still in the school system at this time (see Table 38).

Table 40: Highest Year of Schooling Completed, Newly Arrived (2012-2016) Syrian Youth, South Australia

	Number	Percent
**Year 12 or equivalent**	30	37.0
**Year 11 or equivalent**	9	11.1
**Year 10 or equivalent**	11	13.6
**Year 9 or equivalent**	7	8.6
**Year 8 or below**	24	29.6
**Did not go to school**	0	0.0
**Not stated**	0	0.0
**Total**	81	100.0

Source: ABS, 2016 Census, Usual Place of Address

3.2.3 Iran

The total population from Iran living in South Australia increased from 2,821 persons to 4,521 persons between 2011 and 2016. This represents an average annual growth rate of 9.89% over this time. Youth aged 15-24, who arrived between 2012 and 2016, represented over 40% of all youth at the time of the 2016 Census. They also represented 13.5% of all those who arrived in South Australia between 2012 and 2016 as shown in Table 41.

Table 41: Overview of the Iranian Population, South Australia 2016

Age	Arrived 2012-2016	Total Pop 2016	% Arrived 2012-16
**0-14**	389	536	72.6
**15-24**	258	623	41.4
**25-34**	628	1174	53.5
**35-44**	486	1053	46.2
**45+**	141	1135	12.4
**Total**	1902	4521	42.1

Source: ABS, 2016 Census, Usual Place of Address

One hundred percent of this newly arrived youth population was living in the Greater Adelaide region at the time of the 2016 Census, with half living in the Adelaide-North region as shown in Table 42.

Table 42: Place of Residence for Newly Arrived (2012-2016) Iranian Youth, South Australia

Region	Number	Percent
Greater Adelaide	255	100.0
Rest of State	0	0.0
Total	255	100.0
Adelaide –Central & Hills	53	
Adelaide -North	128	
Adelaide -South	27	
Adelaide -West	35	

Source: ABS, 2016 Census, Usual Place of Address

Most newly arrived youth were not Australian citizens, as shown in Table 43. Almost 78% spoke English well or very well at the time of the 2016 Census and the main languages spoken at home were Persian and Dari. Islam was the main nominated religion by this cohort.

Table 43: Citizenship, Newly Arrived (2012-2016) Iranian Youth, South Australia

Citizenship	Number	Percent
Australian	4	1.6
Not Australian	254	98.4
Not stated	0	0.0
Total	258	100.0

Source: ABS, 2016 Census, Usual Place of Address

Over 80% of newly arrived Iranian youth reported not being in the labour force at the time of the 2016 Census (Table 44) but this is countered by the high proportion who reported being a student at this time (Table 45); most of whom were full time students.

Table 44: Employment Status, Newly Arrived (2012-2016) Iranian Youth, South Australia

	Number	Percent
Not in The Labour Force	219	88.3
In the Labour Force	29	11.7

Source: ABS, 2016 Census, Usual Place of Address

Table 45: Education Participation, Newly Arrived (2012-2016) Iranian Youth, South Australia

FT/PT Student Status	Number	Percent
Not attending	54	22.2
Full-time student	182	74.9
Part-time student	7	2.9
Total (not incl Not Stated)	243	100.0

Source: ABS, 2016 Census, Usual Place of Address

Of those who nominated attending some form of education institution, the majority were attending Secondary School, with 16 persons (just under seven percent) nominating University or some other form of Tertiary education (Table 46). Table 47 shows that just over a third of this cohort had completed Year 12 at the time of the 2016 Census.

Table 46: Type of Educational Institution Attending, Newly Arrived (2012-2016) Iranian Youth, South Australia

	Number	Percent
**Secondary**	130	54.4
**Technical or Further Education**	21	8.8
**University or other Tertiary**	16	6.7
**Other**	9	3.8
**Not Stated**	15	6.3
**Not Applicable**	48	20.1
**Total**	239	100.0

Source: ABS, 2016 Census, Usual Place of Address

Table 47: Highest Year of Schooling Completed, Newly Arrived (2012-2016) Iranian Youth, South Australia

	Number	Percent
**Year 12 or equivalent**	88	34.8
**Year 11 or equivalent**	47	18.6
**Year 10 or equivalent**	58	22.9
**Year 9 or equivalent**	26	10.3
**Year 8 or below**	31	12.3
**Did not go to school**	3	1.2
**Not stated**	0	0.0
**Total**	253	100.0

Source: ABS, 2016 Census, Usual Place of Address

3.2.4 Iraq

The Iraqi population for South Australia increased slightly from the 2011 Census (1,320 persons) to the 2016 Census (1,481); representing an average annual growth rate of 2.33%. Forty youth aged 15 to 24 years were recorded as having arrived between 2012 and 2016. This represented 16% of the total youth population at the time of the 2016 Census and only 12% of all new arrivals from Iraq during this period. As Table 48 shows, most new arrivals during this time were in the 0 to 14 years and 25-34 years age brackets; most likely representing young family groups. One hundred percent of this newly arrived youth cohort were living in the Greater Adelaide region.

Table 48: Overview of the Iraqi Population, South Australia 2016

Age	Arrived 2012-2016	Total Pop 2016	% Arrived 2012-16
**0-14**	90	161	55.9
**15-24**	40	250	16.0
**25-34**	101	340	29.7
**35-44**	55	289	19.0
**45+**	29	441	6.6
**Total**	315	1481	21.3

Source: ABS, 2016 Census, Usual Place of Address

No new arrivals in the 15 to 24 years age bracket were Australian citizens at the 2016 Census and 100% were living in the Greater Adelaide region, the majority in the Adelaide-North area. Almost 94% of this cohort spoke English well or very well, with the main languages spoken at home Arabic and Kurdish. The top response for religion was Islam. Within this cohort 87.5% (21 persons) described themselves as not being in the labour force at the time of the 2016 Census, and as Table 49 shows, over half were also recorded as not having any status as a student.

Table 49: Education Participation, Newly Arrived (2012-2016) Iraqi Youth, South Australia

FT/PT Student Status	Number	Percent
**Not attending**	16	57.1
**Full-time student**	12	42.9
**Part-time student**	0	0.0
**Total (not incl Not Stated)**	28	100.0

Source: ABS, 2016 Census, Usual Place of Address

Numbers for different types of educational setting and levels of education were too low to be considered reliable.

3.2.5 Overview of Refugee Youth Arriving between 2012 and 2016, Middle East Region

The preceding tables in Section 3.2 highlight some of the stark variations within the population birthplace groups making up these four nominated refugee countries of birth, particularly for youth aged 15 to 24 years. For example, the large population numbers arriving from Afghanistan and Iran over this five year inter-censal period – combined new arrivals from these two population groups represent almost 35% of the total population in South Australia from these nominated refugee countries, as shown in Table 50 below.

Youth aged 15 to 24 years in the four Middle Eastern refugee nominated countries comprised just over 21% of the total population, and this age cohort made up 21.2% of all arrivals between 2012 and 2016.

Table 50: Overview of Population for Refugee Nominated Countries across the Middle East Region for South Australia, 2016

	15-24yrs, Arrived 2012-2016	Total Pop 15-24 2016	Total Population	Total Pop Arrived 2012-2016
**Afghanistan**	755	1819	6284	2659
**Syria**	89	127	812	517
**Iran**	258	623	4521	1902
**Iraq**	40	250	1481	315
**Middle East Region**	1142	2819	13098	5393

Source: ABS, 2016 Census, Usual Place of Address

Points to note from the data on youth aged 15 to 24 years from this Middle East region:

- Newly arrived youth in all birthplace groups had small numbers represented in the labour force, but the majority of each cohort, except Iraq, were in secondary school.
- Youth from Afghanistan had the highest proportion living in rural or regional SA (40 persons), but this was still a very low proportion of the total population.
- Afghani youth also had the highest number of Australian citizens (18 persons or 2.4% of this population cohort). Iraq was the only Middle Eastern cohort that had no newly arrived youth with Australian citizenship – although numbers are very small for this cohort.

3.3 Nominated Refugee Countries in the South Asia Region

The four countries making up the nominated refugee birthplace groups for the South Asia region are: Myanmar, Bhutan, Nepal and Pakistan. In 2006 youth aged 15 to 24 years from the four nominated refugee countries in this region made up 34.2% of all refugee youth in Australia and 28.5% in South Australia. By 2016, this group were 57.9% of the national refugee youth population but had also increased to form 61.6% of South Australia's refugee youth population aged 15 to 24 years. This represented an increase of more than seven times the 2006 population for this region, from 493 in 2006 to 3,381 in 2016. These increases appear to be proportionally distributed across all four countries of birth, although the most significant population numbers in 2016 were in the Nepalese and Pakistani populations.

3.3.1 Myanmar

The total population from Myanmar living in South Australia more than doubled in the inter-censal period; increasing from 723 persons to 1,577 persons between 2011 and 2016. This represents an average annual growth rate of 16.88% over this time. Youth aged 15-24, who arrived between 2012 and 2016, represented over 50% of all youth at the time of the 2016 Census as shown in Table 51. They also represented 21.6% of all those who arrived in South Australia between 2012 and 2016.

Table 51: Overview of the Myanmese Population, South Australia 2016

Age	Arrived 2012-2016	Total Pop 2016	% Arrived 2012-16
**0-14**	104	162	64.2
**15-24**	154	304	50.7
**25-34**	242	432	56.0
**35-44**	148	363	40.8
**45+**	66	316	20.9
**Total**	714	1577	45.3

Source: ABS, 2016 Census, Usual Place of Address

The majority of all newly arrived youth from Myanmar were living the Greater Adelaide region at the time of the 2016 Census, with most of them living in the northern region of Adelaide, as shown in Table 52. Almost all (97.5%) of this group were not Australian citizens at the time of the 2016 Census and only 64.5% spoke English well or very well. The main languages spoken at home were Burmese and Chin Haka, the main religion nominated was Christian.

Table 52: Place of Residence for Newly Arrived (2012-2016) Myanmese Youth, South Australia

Region	Number	Percent
Greater Adelaide	148	92.5
Rest of State	12	7.5
Total	160	100.0
Adelaide –Central & Hills	5	
Adelaide -North	110	
Adelaide -South	0	
Adelaide -West	25	

Source: ABS, 2016 Census, Usual Place of Address

Only a small percentage of this group of newly arrived youth were in the labour force at the time of the 2016 Census (see Table 53), but, as Table 54 highlights, a large majority (almost 70%) of this cohort reported being a full-time student. More than half of these students (59 of a possible 108) were in Secondary School and 21 stated they were in University or other tertiary education.

Table 53: Employment Status, Newly Arrived (2012-2016) Myanmese Youth, South Australia

	Number	Percent
Not in The Labour Force	115	87.8
In the Labour Force	16	12.2

Source: ABS, 2016 Census, Usual Place of Address

Table 54: Education Participation, Newly Arrived (2012-2016) Myanmese Youth, South Australia

FT/PT Student Status	Number	Percent
Not attending	38	26.6
Full-time student	105	73.4
Part-time student	0	0.0
Total (not incl Not Stated)	143	100.0

Source: ABS, 2016 Census, Usual Place of Address

Table 55: Type of Educational Institution Attending, Newly Arrived (2012-2016) Myanmarese Youth, South Australia

	Number	Percent
Secondary	59	36.2
Technical or Further Education	14	8.6
University or other Tertiary	21	12.9
Other	14	8.6
Not Stated	17	10.4
Not Applicable	38	23.3
Total	163	100.0

Source: ABS, 2016 Census, Usual Place of Address

3.3.2 Bhutan

The total population of people in South Australian from Bhutan almost doubled from 705 persons in 2011 to a total of 1404 persons in 2016. Youth aged 15 to 24 made up only 3.9% of the total population in 2016, with the majority of the population aged 35+ years. Youth aged 15 to 24 years who had arrived in the 2012 to 2016 period were almost a third of all youth in this age cohort in 2016, but only made up 7.4% of all arrivals in South Australia during this time period.

Table 56: Overview of the Bhutanese Population, South Australia 2016

Age	Arrival 2012-2016	Total Pop 2016	% Arrival 2012-16
**0-14**	8	14	57.1
**15-24**	18	55	32.7
**25-34**	203	479	42.4
**35-44**	169	329	51.4
**45+**	241	527	45.7
**Total**	639	1404	45.5

Source: ABS, 2016 Census, Usual Place of Address

All of these newly arrived youth lived in the Greater Adelaide region in 2016, the majority of them in Adelaide-North. None of this cohort was an Australian Citizen and only 30% spoke English well or not well. The main language spoken at home was Nepali and the main religion was Hinduism.

The population numbers for youth aged 15 to 24 years who arrived between 2012 and 2016 from Bhutan were too small (n=18) for any meaningful data to be drawn from the ABS on employment and education.

3.3.3 Nepal

In 2011 the Nepalese population in South Australia was 1,038 persons, this increased to 2,845 persons by 2016. The represents an average annual growth rate of 22.34%. In 2016 the population cohort of youth aged 15 to 24 years represented 24.6% of the total Nepalese population in South Australia. Youth who had arrived between 2012 and 2016 (474 persons) constituted 67.8% of all youth in this population group and 36.1% of all arrivals from Nepal during this time. One hundred percent of this newly arrived youth population were living in the Greater Adelaide region at the time of the 2016 Census, over half (242 persons) in the Adelaide-North region.

Table 57: Overview of the Nepalese Population, South Australia 2016

Age	Arrived 2012-2016	Total Pop 2016	% Arrived 2012-16
**0-14**	264	459	57.5
**15-24**	474	699	67.8
**25-34**	465	1161	40.1
**35-44**	83	427	19.4
**45+**	24	99	24.2
**Total**	1310	2845	46.0

Source: ABS, 2016 Census, Usual Place of Address

Over 97% of this newly arrived youth cohort were not Australian citizens at the time of the 2016 Census and approximately 83.4% spoke English well or very well. The main language spoken at home was Nepali and the top two religions were Hinduism and Buddhism.

A substantial proportion of newly arrived Nepalese youth aged 15 to 24 years were in the labour force, when compared to other nominated refugee countries in this study (see Table 58 below). A total of 341 newly arrived youth also nominated being a full-time student (Table 59) and a surprisingly large number of these (117) nominated attending University (Table 60). This high number is reflected in the final table, which shows that over 62% of this cohort have completed Year 12 or the equivalent.

Table 58: Employment Status, Newly Arrived (2012-2016) Nepalese Youth, South Australia

	Number	Percent
**Not in The Labour Force**	284	61.1
**In the Labour Force**	181	38.9

Source: ABS, 2016 Census, Usual Place of Address

Table 59: Education Participation, Newly Arrived (2012-2016) Nepalese Youth, South Australia

FT/PT Student Status	Number	Percent
**Not attending**	109	24.0
**Full-time student**	341	75.1
**Part-time student**	4	0.9
**Total (not incl Not Stated)**	454	100.0

Source: ABS, 2016 Census, Usual Place of Address

Table 60: Type of Educational Institution Attending, Newly Arrived (2012-2016) Nepalese Youth, South Australia

	Number	Percent
**Secondary**	98	22.2
**Technical or Further Education**	69	15.6
**University or other Tertiary**	117	26.5
**Other**	27	6.1
**Not Stated**	21	4.8
**Not Applicable**	109	24.7
**Total**	441	100.0

Source: ABS, 2016 Census, Usual Place of Address

Table 61: Highest Year of Schooling Completed, Newly Arrived (2012-2016) Nepalese Youth, South Australia

	Number	Percent
**Year 12 or equivalent**	290	62.1
**Year 11 or equivalent**	58	12.4
**Year 10 or equivalent**	53	11.3
**Year 9 or equivalent**	23	4.9
**Year 8 or below**	36	7.7
**Did not go to school**	7	1.5
**Not stated**	0	0.0
**Total**	467	100.0

Source: ABS, 2016 Census, Usual Place of Address

3.3.4 Pakistan

The overall Pakistani population in South Australia in 2016 was 3,419 people and increase from 1,358 persons in 2011. This equates with an average annual growth rate of 22.34% over this time period. Youth aged 15 to 24 years represented 29.4% of the overall 2016 Pakistan population. Youth who arrived in the 2012 to 2016 period (n=474) represented 67.8% of all youth from Pakistan in 2016 and 15.3% of all arrivals during this 2012 to 2016 time period.

Table 62: Overview of the Pakistani Population, South Australia 2016

Age	Arrived 2012-2016	Total Pop 2016	% Arrived 2012-16
**0-14**	640	871	73.5
**15-24**	305	492	62.0
**25-34**	614	1007	61.0
**35-44**	355	687	51.7
**45+**	73	362	20.2
**Total**	1987	3419	58.1

Source: ABS, 2016 Census, Usual Place of Address

Ninety-six percent of this newly arrived youth cohort were living in the Greater Adelaide region in 2016, with most of this cohort living Adelaide-North. The second largest proportion was living in Adelaide-West. Over 92% of this cohort spoke English well or very well and the main language spoken at home was Pakistani. Islam was the most commonly nominated religion. Only one percent of this cohort (3 people) were Australian citizens at the time of the 2016 Census.

Table 63: Place of Residence for Newly Arrived (2012-2016) Pakistani Youth, South Australia

Region	Number	Percent
**Greater Adelaide**	289	96.0
**Rest of State**	12	4.0
**Total**	301	100.0
**Adelaide –Central & Hills**	36	
**Adelaide -North**	131	
**Adelaide -South**	45	
**Adelaide -West**	80	

Source: ABS, 2016 Census, Usual Place of Address

Just over 30% of this newly arrived youth cohort were in the labour force at the time of the 2016 Census (Table 64). However, this data needs to be contextualised by examining Table 65 where we can see that almost 74% of this cohort nominated being a student in 2016 (71.3% of those full time). Table 66 shows that, unlike other youth cohorts in these nominated refugee countries, the majority of this student population group (35.6%) were at University or another form of tertiary institution; followed closely by students at Secondary School (27.8%).

Table 64: Employment Status, Newly Arrived (2012-2016) Pakistani Youth, South Australia

	Number	Percent
**Not in The Labour Force**	181	69.1
**In the Labour Force**	81	30.9

Source: ABS, 2016 Census, Usual Place of Address

Table 65: Education Participation, Newly Arrived (2012-2016) Pakistani Youth, South Australia

FT/PT Student Status	Number	Percent
**Not attending**	73	26.2
**Full-time student**	199	71.3
**Part-time student**	7	2.5
**Total (not incl Not Stated)**	279	100.0

Source: ABS, 2016 Census, Usual Place of Address

Table 66: Type of Educational Institution Attending Newly Arrived (2012-2016) Pakistani Youth, South Australia

	Number	Percent
**Secondary**	79	27.8
**Technical or Further Education**	12	4.2
**University or other Tertiary**	101	35.6
**Other**	4	1.4
**Not Stated**	15	5.3
**Not Applicable**	73	25.7
**Total**	284	100.0

Source: ABS, 2016 Census, Usual Place of Address

3.3.5 Overview of Refugee Youth Arriving between 2012 and 2016, South Asia Region

The preceding tables in Section 3.3 highlight the four population birthplace groups making up the South Asia region – Myanmar, Bhutan, Nepal and Pakistan focusing particularly on youth aged 15 to 24 years. Table 67 highlights the dominance of the Nepalese and Pakistani birthplace groups in this age cohort. As such, these two population groups combined makeup 81.9% of all youth arriving from 2012 to 2016 and 76.8% of the total youth population in 2016 in these selected South Asian refugee population groups.

Youth aged 15 to 24 years in the four South Asian refugee nominated countries comprised just over 16% of the total population, and this age cohort made up 20.4% of all arrivals between 2012 and 2016.

Table 67: Overview of Population for Refugee Nominated Countries across the South Asia Region for South Australia, 2016

	15-24yrs, Arrived 2012-2016	Total Pop 15-24 2016	Total Population	Total Pop Arrived 2012-2016
**Myanmar**	154	304	1577	714
**Bhutan**	18	55	1404	639
**Nepal**	474	699	2845	1310
**Pakistan**	305	492	3419	1987
**South Asia Region**	951	1550	9245	4650

Source: ABS, 2016 Census, Usual Place of Address

Points of note from the data on youth aged 15 to 24 years from this South Asia region:

- The Pakistani and Nepalese youth levels of education appear to be significant outliers across all nominated refugee countries with over 100 students nominating University as their education setting. This may just indicate that the Census data is picking up international students attending University as well as a refugee population.

- Newly arrived Pakistani and Nepalese youth also showed much higher rates of labour force participation – 30.9% and 38.9% respectively.

- Bhutan had the lowest participation rates in education across all of the nominated refugee countries in this study – with only 35.3% of this newly arrived youth cohort. It also had no one nominated as being in the labour market. However, it must be remembered that population numbers were relatively small in this birthplace group.

4. Key points from all birthplace groups

- Citizenship levels were very low among all nominated refugee birthplace groups. However, it must be remembered that this report only looked at youth who had arrived between 2012 and 2016 and their citizenship status as at the 2016 Census. Citizenship processes take a considerable amount of time and are often not the first priority for this group (some may not even be eligible for quite a period of time), so it is not unexpected to see such low citizenship levels.

- The proportion of each newly arrived youth population cohort in some form of schooling varied considerably across birthplace groups: from a low 35.3% and 42.9% respectively in Bhutan and Iraq to very high levels in Syria and Liberia (100% and 93% respectively). Considering all young people under the age of 17 are required to attend full-time schooling, training or work for at least 25 hours per week, some of these proportions seem low.

- Levels of participation in work were low overall – seven of the 15 nominated refugee countries had labour force participation rates below 10%. However, this also varied considerably across nominated refugee birthplace groups: from zero percent participation in Bhutan, Democratic Republic of Congo, Sudan and South Sudan to a high of 39.7% for Liberia and 38.9% for Nepal. However, as with education – many of these birthplace group percentages are based on small population numbers and can be considered unreliable.

- In all of the preceding statistics it must be remembered that the Census data captures all persons from those birthplace groups within the nominated age range and arrival dates. This means that it captures individuals in these population groups who may not have arrived with a refugee status. This includes, potentially, skilled (non-refugee status) migrants and international students from these countries and this may help to explain the unusually high proportions of students from Pakistan and Nepal who were in University at the time of the 2016 Census compared to other countries in this study.

C. Refugee Youth 15-24 years –South Australia: Survey Data

This Section reports on several categories of primary data collected from the completed survey with 630 refugee background youth residing in South Australia between the periods of 2016 and 2018. As indicated in the 'Selection Criteria', unlike the secondary data, all youth who participated in the survey reported arriving in Australia or that their parents arrived in Australia, on a humanitarian (refugee) visa. The corresponding visa categories—as identified by participants—included: subclass 200 (Refugee), 201 (In–Country Special Humanitarian Program), 202 (Global Special Humanitarian Program), 203 (Emergency Rescue) and 204 (Woman at Risk), although this list is not exhaustive. The country of birth of principal visa applicants is applied to secondary visa applicants.

Hence unlike the secondary data in Section B of this Report, survey data excluded all youth who had arrived in Australia on non-humanitarian visas, for example international students and skilled migrants. The primary data pertaining to the survey sample are presented according to the following categories:

1. Cultural and demographic background
2. Gender
3. Age
4. Length of stay in Australia
5. Refugee camp experience
6. Australian detention experience
7. Previous trauma experience
8. Employment status and nature
9. Attending educational institution

1. Cultural and Demographic Background of Participants

Table 68: Cultural / Ethnic Background of Survey Participants

Country	Frequency	Percent
Afghan	159	25.2
Bhutanese	31	4.9
Burmese	61	9.7
Congolese	67	10.6
Ethiopian	28	4.4
Iranian / Persian	15	2.4
Iraqi	20	3.2
Nepali	43	6.8
Pakistani	12	1.9
Somali	7	1.1
Sudanese	64	10.2
Syrian	40	6.3
Liberian	26	4.1
Sierra Leonean	2	0.3
Burundian	9	1.4
Eritrean	9	1.4
Rwandan	9	1.4
Sri Lankan	3	0.5
Ghanaian	5	0.8
Others African	15	2.4
Others Middle East	2	0.3
Others South Asia	3	0.5
Total	630	100.0

Notes:

- Re cultural/ethnic background, most participants identified as being from Afghanistan (25.2%); Congo (10.6%); Sudan (10.2%) and Burma / Myanmar (9.7%).
- No distinction was made between Dem. Rep. of Congo and Rep. of Congo nor Sudan and South Sudan.
- The relatively large sample from Afghanistan together with the initial decision to include Afghanistan within the migration region of the 'Middle East', and not 'South Asia', could likely be the reason for the slightly disproportionate sample sizes from the three regions (Table 69).

Table 69: Participants' Migration by Region

Region	Frequency	Percent
South Asia	153	24.3
Middle East	236	37.5
Africa	241	38.3
Total	630	100.0

Table 70: Participants' Gender

		Frequency	Percent
	Male	255	40.5
	Female	375	59.5
	Total	630	100.0

Table 71: Participants' Age

Age in Years	Frequency	Percent
14	7	1.1
15	56	8.9
16	70	11.1
17	81	12.9
18	78	12.4
19	93	14.8
20	66	10.5
21	55	8.7
22	54	8.6
23	32	5.1
24	20	3.2
25	14	2.2
26	4	0.6
**Total**	630	100.0

Table 72: Participants' Age: Over vs. Under 18.

		Frequency	Percent
	Under18	214	34.0
	Over18	416	66.0
	Total	630	100.0

Figure 5: Participants' Age

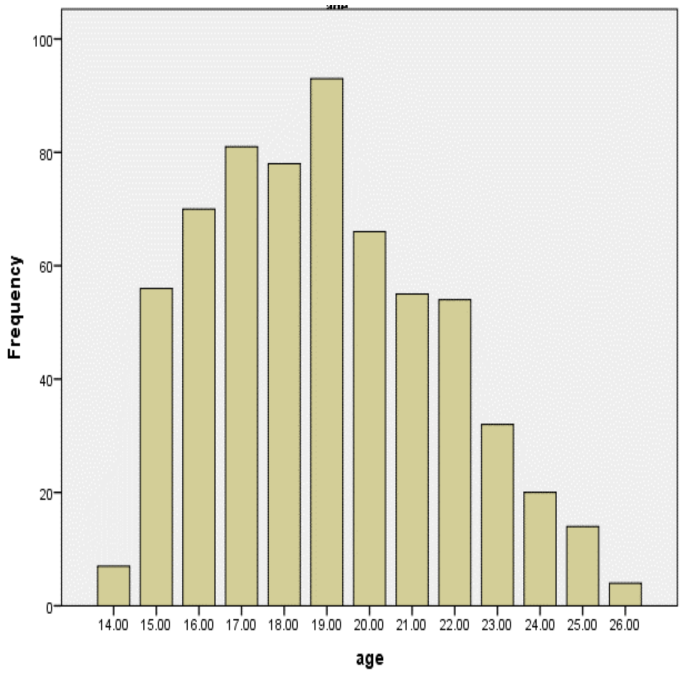

Years

Notes:

- There were twice as many participants over 18 years (66%) than those under 18 years (34%). This is understandable because 'under 18' included 3 age groups (14, 15 & 17 year-olds) while 'over 18' included 7 age groups (18 to 24 years). In addition, researchers were more willing to include participants over 24 years (up to 26 years) than to include participants less than 15 years because the survey was deemed less suitable for those under 15 years.
- The 7 participants in the '14 years' category (Table 71) were 1-3 months short of 15 years.
- Most participants were between 17 and 19 years old (40.1%).

Table 73: Length of Residency in Australia

Number of Years	Frequency	Percent
.00	11	1.7
1	94	14.9
2	66	10.5
3	41	6.5
4	72	11.4
5	42	6.7
6	45	7.1
7	45	7.1
8	46	7.3
9	46	7.3
10	43	6.8
11	30	4.8
12	24	3.8
13	11	1.7
14	5	.8
15	3	.5
16	2	.3
17	1	.2
18	1	.2
20	1	.2
Total	629	99.8

Figure 6: Length of Residency in Australia

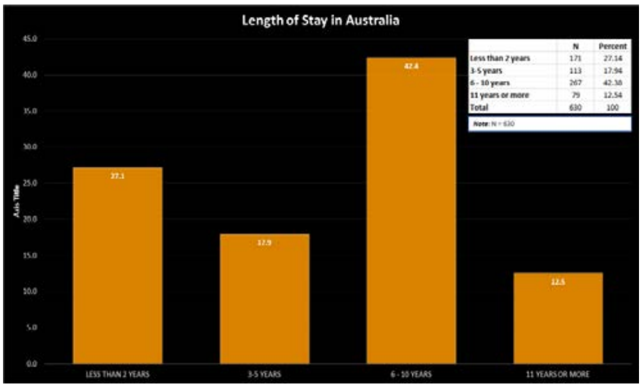

Length of residency in Australia (years)

Notes:

- Most participants had resided between 6 and 10 years in Australia (267; 42.4%). There were 171 (27.1%) participants who had lived between 1-2 years in Australia, 113 (17.9%) between 3-5 years, and 78 (12.4%) had resided more than 11 years in Australia.
- Australian Residency of at least 1 year was a project criterion because the literature is well established that the initial months after migration is an especially stressful time for most refugee populations, who are engaged in finding accommodation, learning English and trying to grasp vast amounts of information about new and unfamiliar systems during that time.
- During the initial months after migration, people are usually inundated with appointments at various institutions, agencies and with service providers (i.e. Centrelink, settlement service agencies, schools, medical clinics) that they are less suitable as participants in this type of research project, during this time period. Nonetheless, there were a few participants who had lived between 10-12 months in Australia (11; 1.7%).
- As aforementioned in the Introduction to this Report, initial participant selection criteria required participants to have lived in Australia up to 10 years, because the literature indicates that after about a decade in the host country, refugee populations can be considered adequately 'settled' to become part of the general, mainstream population. However, after feedback from Bilingual Youth Workers (BYWs) assisting with survey completion that this assumption was not completely accurate in relation to youth in their communities, this project criterion was extended to '15 years residency in Australia'.
- There were 5 (0.9%) participants who had lived more than 15 years in Australia.

Table 74: Participants' Employment Status

	Frequency	Percent
**Yes**	116	18.4
**No**	514	81.6
**Total**	630	100.0

Table 75: Participants' Main Activity

	Frequency	Percent
**High School**	340	53.9
**TAFE**	79	12.5
**University**	134	21.3
**2 or more institutions**	12	1.9
**Other**	59	9.7
**Total**	624	99.0

Notes:

- No survey item directly inquired about the nature of participants' educational institution. This omission was due to the added precaution of ensuring confidentiality to participants who might have felt uncomfortable to describe their education institution; and for the peace of mind of institutional authorities.

- Instead, an indirect question inquired how participants spent most of their time, with an assumption that students would spend most of their time at an educational institution. From the responses to this item, information regarding participants' educational institution was obtained. However, some participants had spent more time on activities other than educational activities, explaining the relatively high number of responses belonging to the 'other' category (almost 10%) including those who did not mention an educational institution; instead mentioning an activity such as caring for siblings or grandparents, employment, and household chores (Table 75).

- Less than 20% of the sample (116) were engaged in some form of employment. Of them, 62 (53.4%) were engaged in casual/temporary work and 42 (25%) were in permanent full-time or part-time employment (Table 74).

2. Participant's Life Course Experiences before or immediately after Arriving in Australia

Table 76: Participants' Refugee Camp Experience

	Frequency	Percent
**Yes**	298	47.3
**No**	328	52.1
**Total**	626	99.4

Table 77: Participants' Detention Camp Experience

	Frequency	Percent
**Yes**	55	8.7
**No**	565	89.7
**Total**	620	98.4

Notes.

- Participants' life course experiences such as living in a refugee camp before arriving in Australia or in a detention camp immediately after arriving in Australia as well as, past traumatic experiences are likely to impact on their psychosocial wellbeing and influence their settlement outcomes. Tables 76 and 77 afford data relating to these experiences. It is noteworthy that almost half of the participants surveyed (47.3%) admitted experiencing life in a refugee camp indicating that it is likely that they would have experienced at least some of the traumatic events listed in Table 78.

- Survey Question 25 inquired about participants' previous traumatic experiences. A potentially interesting finding was that less than 18% of participants reported experiencing no pre-migration trauma (n=112; 17.8%) (Table 78). The traumatic events mostly identified included: lack of food and water (25%), witnessing someone being badly injured or killed (16%), and other extremely stressful events (17%). This information gives us a good idea of the challenges encountered by refugee background youth, over and beyond that of youth belonging to the general population in Australia.

- Two high schools refused consent for their students/participants to complete Question 25 (requiring us to paste a blank piece of paper over Q25) because of teachers' concerns that answering or perusing Q25 might be distressing for their students. However almost 95% (594) of participants had answered this Survey item including 142 (22.5%) who said: they *'Do not wish to answer'*.

Table 78: Participants' Previous Trauma Experiences

Event	n	%	Event	n	%
**Lack of food or water**	157	24.9%	**Brain washing or re-schooling (being forced to change your beliefs)**	21	3.3%
**Witnessing someone being badly injured or killed**	103	16.3%	**Combat situation (war)**	48	7.6%
**Other extremely stressful events**	108	17.1%	**Being threatened with a weapon, held captive or kidnapped**	27	4.3%
**Fire, flood or other natural disaster**	96	15.2%	**Ill health without access to medical care**	70	11.1%
**Being close to death**	74	11.7%	**Being physically attacked or assaulted**	60	9.5%
**Life threatening accident**	58	9.2%	**Lack of shelter**	80	12.7%
**Being put in prison/locked up by the police/government**	15	2.4%	**Forced isolation**	29	4.6%
**Forced separation from family members**	55	8.7%	**Witnessing rape or sexual abuse**	19	3.0%
**Threatening events happening to someone close to you**	91	14.4%	**Being tortured or the victim of terrorists**	9	1.4%
**Sustaining a serious injury**	27	4.3%	**Rape/ Sexual molestation**	16	2.5%
**Murder of strangers or a stranger**	24	3.8%	**None of the above**	112	17.8%
**The death of a family member or friend from unnatural causes (e.g. accident, suicide, murder, drug abuse, etc.)**	83	13.2%	**Do not wish to answer**	142	22.5%

References

Australian Bureau of Statistics (ABS). (2016). *Census of population and housing*.

Australian Government. (2010). National Strategy for Young Australians. Retrieved from http://www.youthpolicy.org/national/Australia_2010_National_Youth_Strategy.pdf

Berry, J. W. (1997). Immigration, Acculturation, and Adaptation. *Applied Psychology, 46*(1), 5-34. doi:10.1111/j.1464-0597.1997.tb01087.x

Berry, J. W. (2011). Integration and multiculturalism: Ways towards social solidarity. *Papers on Social Representations, 20*(1), 2.1-2.21.

Birman, D., & Trickett, E. J. (2001). Cultural transitions in first-generation immigrants: Acculturation of Soviet Jewish refugee adolescents and parents. *Journal of cross-cultural psychology, 32*(4), 456-477.

Cassity, E. A. (2007). Voices shaping education: Young African refugees in Western Sydney high schools. *International Education Journal, 8*(3), 91-104.

Cassity, E. A. (2013). Voices shaping education: Young African refugees in Western Sydney high schools. *International Education Journal: Comparative Perspectives, 8*(3).

Colic-Peisker, V. (2009). Visibility, settlement success and life satisfaction in three refugee communities in Australia. *Ethnicities, 9*(2), 175-199. doi:10.1177/1468796809103459

Dandy, J., Dunn, K., Jetten, J., Paradies, Y., Robinson, L., & Ziaian, T. (2017). Intercultural relations in Australia In J. W. Berry (Ed.), *Mutual Intercultural Relations*. Cambridge, GB: Cambridge University Press

de Anstiss, H., Ziaian, T., Procter, N., Warland, J., & Baghurst, P. (2009). Help-seeking for mental health problems in young refugees: a review of the literature with implications for policy, practice, and research. *Transcultural psychiatry, 46*(4), 584-607.

Department of Education and Early Childhood Development. (2011). Refugee Status Report. Retrieved from http://www.education.vic.gov.au/about/research/Pages/reportdatarefugee.aspx

Department of Home Affairs (DOHA). (2018). Humanitarian Programme figures. Retrieved from https://www.homeaffairs.gov.au/about/corporate/information/fact-sheets/60refugee

Department of Immigration and Citizenship (DIAC). (2009). Refugee and humanitarian issues: Australia's response. Retrieved from http://www.immi.gov.au/media/publications/refugee/ref-hum-issues/ref-hum-issues-june09.htm

Department of Immigration and Multicultural Affairs (DIMA). (2007). Settlement target group arrivals: July-December 2006. . Retrieved from http://www.immi.gov.au/living-in-australia/delivering-assistance/government-

Graham, H. R., Minhas, R. S., & Paxton, G. (2016). Learning problems in children of refugee background: a systematic review. *Pediatrics, 137*(6), e20153994.

Hatoss, A., O'Neill, S., & Eacersall, D. (2012). Career choices: Linguistic and educational socialization of Sudanese-background high-school students in Australia. *Linguistics and Education, 23*(1), 16-30. doi:10.1016/j.linged.2011.10.003

Hugo, G. (2010). *An overview of migration trends and developments for South Australia, Australia and globally. Presentation to the 2011 Migration Update Conference, Adelaide*. Retrieved from

Hugo, G., McDougall, K., Tan, G., & Feist, H. (2014). *CALD Youth Census Report 2014*. Retrieved from South Austraila: Adelaide:

Hugo, G., Vas Dev, S., Wall, J., Young, M., Sharma, V., & Parker, K. (2011). Economic, social and civic contributions of first and second generation humanitarian entrants: Final report.

Kovacev, L., & Shute, R. (2004). Acculturation and social support in relation to psychosocial adjustment of adolescent refugees resettled in Australia. *International Journal of Behavioral Development, 28*(3), 259-267.

Matthews, J. (2008). Schooling and settlement: Refugee education in Australia. *International Studies in Sociology of Education, 18*(1), 31-45.

Miller, E., Ziaian, T., & Esterman, A. ((2017)). Australian school practices and the education experiences of students with a refugee background: A review of the literature. *International Journal of Inclusive Education*, 1-21. doi:10.1080/13603116.2017.1365955

Miller, J., Mitchell, J., & Brown, J. (2005). African refugees with interrupted schooling in the high school mainstream: Dilemmas for teachers.

Minza, W. M. (2012). Young migrants and education-to-work transitions in Pontianak, West Kalimantan. *The Asia Pacific Journal of Anthropology, 13*(1), 64-75.

Rah, Y., Choi, S., & Nguyễn, T. S. T. (2009). Building bridges between refugee parents and schools. *International Journal of Leadership in Education, 12*(4), 347-365.

Refugee Council of Australia. (2010). Economic,Civic and Social Contributions of Refugees and Humanitarian Entrants: A Literature Review. Prepared for the Department of Immigration and Citizenship, released February 2010.

Renzaho, A., McCabe, M., & Sainsbury, W. J. (2011). Parenting, role reversals and the preservation of cultural values among Arabic speaking migrant families in Melbourne, Australia. *International journal of intercultural relations, 35*(4), 416-424. doi:10.1016/j.ijintrel.2010.09.001

Taylor, S., & Sidhu, R. K. (2012). Supporting refugee students in schools: what constitutes inclusive education? *International Journal of Inclusive Education, 16*(1), 39-56.

Ziaian, T., de Anstiss, H., Antoniou, G., Baghurst, P., & Sawyer, M. (2012). Resilience and its association with depression, emotional and behavioural problems, and mental health service utilisation among refugee adolescents living in South Australia. *International Journal of Population Research, 2012*.

Ziaian, T., de Anstiss, H., Antoniou, G., Baghurst, P., & Sawyer, M. (2013). Emotional and behavioural problems among refugee children and adolescents living in South Australia. *Australian Psychologist, 48*(2), 139-148.

Ziaian, T., Puvimanasinghe, T., Miller, E., de Anstiss, H., Dollard, M., Esterman, A., . . . Afsharian, A. J. (2018). Challenges, facilitators and enablers of conducting research with youth from refugee backgrounds. *The Australian Community Psychologist, 29*(2), 56.

University of
South Australia
